# 3-O-Acetyl-11-Keto-**β**-Boswellic Acid Suppresses Colitis-Associated Colorectal Cancer by Inhibiting the NF-Kb Signaling Pathway and Remodeling Gut Microbiota

**DOI:** 10.32604/or.2025.062386

**Published:** 2025-07-18

**Authors:** Fang Xu, Wan Li, Xiang-Jin Zheng, Yue Hao, Yi-Hui Yang, Hong Yang, Sen Zhang, Wan-Xin Cao, Xiao-Xue Li, Xu Zhang, Guan-Hua Du, Teng-Fei Ji, Jin-Hua Wang

**Affiliations:** 1 The State Key Laboratory of Bioactive Substance and Function of Natural Medicines, Institute of Materia Medica, Chinese Academy of Medical Science and Peking Union Medical College, Beijing, 100050, China; 2 Beijing Key Laboratory of Innovative Drug Discovery and Polymorphic Druggability Research for Cerebrovascular Diseases, Chinese Academy of Medical Science and Peking Union Medical College, Beijing, 100050, China

**Keywords:** 3-O-Acetyl-11-keto-b-boswellic acid (AKBA), colorectal cancer (CRC), inflammation, gut microbial, NF-κB pathway

## Abstract

**Objectives:**

Colorectal cancer (CRC) is one of the most common cancers all over the world. The progression of CRC is associated with inflammation and disruptions in intestinal flora. 3-O-Acetyl-11-keto-β-boswellic acid (AKBA) has been noted for its potent anti-inflammatory properties. However, the effect of AKBA on colon cancer caused by inflammation and its mechanism are not unclear. The study is to explore the effect of AKBA on CRC and its mechanism.

**Materials and Methods:**

Cell proliferation, (5-ethynyl-2^′^-deoxyuridine, EdU)-DNA synthesis assay and colony formation were used to assess the effect of AKBA on the proliferation of CRC cells. Flow cytometry was employed to analyze the cell cycle and apoptosis rate of cells treated with AKBA. RNA sequencing was done to explore the underlying mechanisms of AKBA. Western blot was used to assess the expression of key proteins in the nuclear factor kappa-B (NF-κB) signaling pathway after the treatment of AKBA. Real-time quantitative PCR (RT-qPCR), enzyme-linked immunosorbent assay (ELISA), and Meso Scale Discovery (MSD) assays were employed to check the anti-inflammation effects of AKBA on Lipopolysaccharide (LPS)-induced RAW264.7 cells and LPS-induced mouse model. Additionally, the Azoxymethane/Dextran sulfate sodium (AOM/DSS)-induced colitis-associated CRC model was used to evaluate the anti-CRC effect of AKBA. Gut microbiota profiling of fecal samples from CRC mice, both with and without AKBA treatment, was conducted through metagenomic sequencing analysis.

**Results:**

Our results showed that AKBA reduced the proliferation of HCT116 and SW620 cells, increased apoptosis of cells, and arrested the cell cycle at the G2/M phase. Results from RNA-seq showed that AKBA inhibited CRC by inhibiting the NF-κB signaling pathway and reducing cellular inflammation. Furthermore, AKBA reduced the levels of inflammatory cytokines, including tumor necrosis factor-α (TNF-α), Interferon-γ (IFN-γ), Interleukin-IL-12p70 (IL-12p70), Interleukin-1β (IL-1β), and tumor necrosis factor-α (TNF-α) in both the spleen and serum of LPS-induced acute inflammation mice. Additionally, AKBA inhibited the development of AOM/DSS-induced colitis-associated colon cancer in mice and positively influenced gut microbiota.

**Conclusion:**

This study highlights the inhibitory effect of AKBA on colitis-associated CRC and reveals a novel aspect of its role in the remodeling of gut microbiota. These findings suggest that AKBA may be used as a potential therapeutic agent for CRC.

## 1 Introduction

Colorectal cancer (CRC) is one of the most common cancers all over the world, ranking 3rd in terms of incidence (9.6%) and 2nd in terms of mortality (9.3%) [1]. Both hereditary and environmental risk factors promote the development of CRC [2], while conditions like ulcerative colitis (UC) and imbalance in gut microbiota also increase the risk for the initiation and progression of this disease [3,4].

The treatment of CRC faces several challenges. Early detection is difficult as the disease often develops without clear symptoms. Although surgery is a common treatment, it may not completely eradicate the cancer and can lead to complications. Chemotherapy can cause side effects and lead to drug resistance. Although targeted therapies and immunotherapy have shown some promises, their overall effectiveness remains limited. Moreover, the treatment of CRC has imposed a substantial economic burden on both society and family [5]. Therefore, there is a crucial need to find out new ways for the treatment of colon cancer.

Natural products are the major resources of new drugs, which have few side effects. Acetyl-11-keto-β-boswellic acid (AKBA) is extracted from the gum resin of frankincense trees [6]. This natural product is widely used to treat inflammatory illnesses including arthritis, colitis, and asthma as well as some diseases in Africa, India, and China. In recent years, AKBA has been found to trigger apoptosis and suppress tumor growth in various cancer cell lines, including CRC [7], gastric cancer cells [6,8], and glioblastoma [9,10]. Additionally, AKBA has been found to modulate the inflammatory response and provide defense in DSS-induced colitis by modulating the NF-κB and JNK-p38/MAPK signaling pathways [11]. While previous research mainly focused on AKBA’s anti-inflammatory or anti-tumor properties, its effects on colitis-associated colorectal cancer (CAC) and their mechanisms are still unclear.

NF-κB signaling pathway plays an important role in biological activities, such as inflammation, immune responses, proliferation, cell survival and so on [12,13]. Abnormal activation of this pathway is frequently associated with the development of many cancers, including CAC [14,15]. This pathway is known to promote the level of pro-inflammatory cytokines, anti-apoptotic genes, and cell proliferation-related genes, thus contributing to tumor initiation, progression, and resistance to therapy [16,17]. Therefore, inhibiting the NF-κB pathway has emerged as a promising strategy for cancer treatment, particularly in the context of CAC where inflammation acts as an important role in it.

In addition to cellular signaling pathways, the gut microbiota has recently attracted more interest in cancer research [18]. This complex community of microorganisms inhabiting the gastrointestinal tract has been found to affect the physiology and pathophysiology of the host. An imbalance in the gut microbiota was associated with inflammatory bowel diseases and CRC. More new evidences indicated that the gut microbiota can influence the host’s immunity, affect intestinal inflammation, and interact with cancer cells, is related to the onset and progression of CRC [19,20]. As a result, altering the gut microbiota emerged as a promising approach for preventing and treating CRC.

In this study, we investigated the effect of AKBA on CRC progression by inhibiting the NF-κB pathway and modulating the gut microbiota. Our results showed that AKBA may exert its anti-tumor effects by dual mechanisms: directly targeting the NF-κB pathway and indirectly influencing the gut microbiota composition. Based on the above results, we think that our findings may provide valuable insights into developing innovative therapeutic strategies for preventing and treating CAC.

## 2 Materials and Methods

### 2.1 Chemotherapeutic Drug

AKBA was isolated from the resin seeping (Olibanum) from the bark of *Boswellia carterii* Birdw. The olibanum medicinal materials were from Grand Bazaar in Urumqi City, Xinjiang, China, and were identified as olibanum from *Boswellia carteri* Birdw by Prof. Tengfei Ji. Purified AKBA was a colorless and crystalline compound, the molecular formula was C_32_H_48_O_5_ and the formula weight was 512.4, purity was greater than 98%, the HR-ESI-MS spectrum (Fig. S1), ^1^H NMR (Fig. S2) and ^13^C NMR (Fig. S3) spectrum were provided by Prof. Tengfei Ji. AKBA was dissolved in Dimethyl sulfoxide (DMSO, MP Biomedicals Co., Ltd., 196055, Solon, OH, USA) at 100 mM as a stock solution, and stored at −20°C for use in cells. The final concentration of DMSO in cell culture was no more than 0.1%. For the animal experiment, AKBA was suspended with 0.5% carboxymethylcellulose (CMC-Na, Solarbio Life Science Co., Ltd., C8620, Beijing, China) at 9 mg/mL as stock solution.

### 2.2 Cell Culture

Human colorectal carcinoma HCT116, SW620 cell lines, RAW 264.7 cells (mouse leukemia cells of monocyte-macrophage) were obtained from the Procell (Pricella Life Science & Technology Co., Ltd., Wuhan, China). Cells were cultured in RPMI-1640 medium (Pricella Life Science & Technology, PM150110) containing 10% fetal bovine serum (FBS, v/v, Pricella Life Science & Technology, 164210-50) and 1% penicillin/streptomycin (Solarbio, P1400). Cells were maintained at 37°C in a humidified atmosphere with 5% CO_2_ in a CO_2_ incubator (Hera cell Vios 160I, Thero Scientific, Munich, Germany). Cells within 10 passages and tested negative for mycoplasma contamination (Myco-Lumi^TM^ Detection kit for mycoplasma by luminescence method, Beyotime Biotechnology Co., Ltd., C0298S, Shanghai, China) were used for the experiments.

### 2.3 Cell Proliferation

Cell proliferation was checked by the CCK-8 assay after AKBA treatment. Cells were seeded in 96-well plates at 3 × 10³ cells/well (Beaver Biomedical Engineering Co., Ltd., Suzhou, China) and treated with 0–100 µM AKBA for 24, 48, and 72 h. The CCK-8 assay was done according to user manual.

### 2.4 EdU-DNA Synthesis Assay

Cell-Light EdU Apollo 567 *In Vitro* kit (RIBOBIO Co. Ltd., C10310-1, Guangzhou, China) was used to check DNA synthesis activity in HCT116 and SW620 cells treated with 0, 20, 30, and 40 µM AKBA for 24 h. Briefly, 50 μM 5-ethynyl-2^′^-deoxyuridine (EdU) solution was added and incubated for 2 h, followed by fixation with 4% paraformaldehyde and permeabilization by 0.5% Triton X-100. Apollo stain reaction solution was used to stain cells. Cells were counter-stained by Hoechst 33342. EdU-positive cells were imaged and quantified. Data were obtained in triplicate.

### 2.5 Plate Colony Formation Assay

The plate cloning assay was done to assess the growth ability in 2D dimension of HCT116 and SW620 cells treated with 0, 20, 30, and 40 μM AKBA. Cells were seeded at 500 cells/well in 6-well plates and incubated for 24 h before AKBA treatment. After 14 days, cells were fixed with 4% paraformaldehyde (Solarbio, P1110) and stained with 0.1% crystal violet solution (Solarbio, G1063). The experiment was performed in triplicate.

### 2.6 Cell Cycle Assay

Cells (2 × 10^6^) were seeded in 6 cm dishes, treated with 20, 30, and 40 μM AKBA for 24 h. Cells were harvested, fixed in 70% ethanol at −4°C for 12 h, then stained with RNase A (20 µg/mL) and Propidium iodide PI (50 μg/mL). Fluorescence was analyzed using a C6 flow cytometer at 488 nm, with data processed using FlowJo 10.8.1 software.

### 2.7 Cell Apoptosis

Cells (2 × 10^6^) were planted into 6 cm dishes and treated with 20, 30, and 40 μM AKBA for 24 h. The detailed experimental methods were described previously [21].

### 2.8 mRNA Transcriptome Sequencing and Data Analysis

To explore the anti-tumor mechanism of AKBA, differentially expressed genes (DEGs) were detected via mRNA transcriptome sequencing in HCT116 and SW620 cells that had been exposed 30 µM AKBA for 48 h. The detailed methods and procedures were described previously [21]. DEGs were screened using the following criteria |log_2_(Fold Change)| ≥ 3 and adjusted *p* value ≤ 0.05.

### 2.9 RNA Extraction and Real-Time Quantitative PCR

Cells were plated at a density of 3 × 10^5^ cells per well and allowed to incubate for 24 h. They were then stimulated with 1 µg/mL LPS (Solarbio, L8880) for 1 h and subsequently treated with 20, 30, and 40 µM AKBA for 24 h. The RNA extraction, reverse transcription, PCR, and quantitative methods were described previously [22]. The primer sequences for important cytokines (IFN-γ, IL-1β, IL-6, TNF-α, and IL-10) are listed in Table S1.

### 2.10 Cellular Inflammation Model and Inflammatory Cytokine Assays

RAW 264.7 cells were seeded at 3 × 10^5^ cells/well in 6-well plates, incubated for 24 h, stimulated with 1 µg/mL LPS for 1 h, and treated with 20, 30, and 40 µM AKBA for 24 and 48 h. Supernatants were collected, centrifuged, and analyzed using ELISA kits to measure cytokine concentrations IL-1β (the mouse IL-1β ELISA kit, Biocreative, Shenzhen, China), IL-6 (4A biotech, Suzhou, China) and TNF-α (4A Biotech, Suzhou, China).

### 2.11 Western Blotting Assay

Cells were lysed with RIPA buffer (Beijing applygen Co., Ltd., C1053, Beijing, China) containing protease (Applygen, P1265) and phosphatase inhibitors (Applygen, P1260). Protein concentration was determined using a BCA kit (Beyotime, P0009, Shanghai, China). A total of 20–50 µg of protein was separated using 10% SDS-PAGE and then transferred to a PVDF membrane. The membrane was blocked with 5% skim milk in TBS-T and incubated with primary antibodies overnight at 4°C. Following washing, the membrane was treated with secondary antibodies (1:5000) for 1 h at room temperature. Protein bands were detected using a BeyoECL Plus kit (Beyotime, P0018M) and imaged with a chemiluminescence system (Shanghai Tanon Technology Co., Ltd., 5200, Shanghai, China). Details of the primary antibodies can be found in Table S2. BMS345541 (MCE, HY-10519) was utilized as an IKK inhibitor.

### 2.12 Animals

BALB/c mice were obtained from Beijing Vital River Laboratory Animal Technology Co., Ltd. (Beijing, China). The mice, approximately seven weeks old and weighing between 18 to 20 g, were used in this study. The breeding conditions of the mice were described previously [10], and the ethical considerations are detailed in the Ethics Approval section. The number of animal experimental ethical inspections is No. 00005266.

### 2.13 Establishment of LPS-Induced Inflammation Model in Mice

The body weight of BALB/c male mice was 18–20 g and acclimated for 3 days before the experiment then the 30 mice were randomized into the following groups, normal, LPS (5 mg/kg) model, dexamethasone (DXM, 5 mg/kg, Solarbio, D6950) as positive control group, and AKBA groups (30 and 90 mg/kg). Each group was continuously gavaged administration for 6 d. The control group and model group were given equal volumes of 0.05% CMC-Na (Solarbio, IS9000), 0.1 mL/10 g. Weight changes of mice were recorded daily. On day 7th, mice received LPS (5 mg/kg) to prepare the acute inflammation model except for the normal control group, and the volume of intraperitoneal injection was 0.1 mL/10 g. After 6 h, blood and spleen were collected.

### 2.14 Establishment of AOM/DSS-Induced Colitis-Associated Crc Model in Mice

The AOM/DSS-induced colitis-associated CRC model is currently the most widely used model for the chemical induction of CRC [23]. 50 male BALB/c mice were adaptively fed for 5 days. Azoxymethane (AOM, Sigma Aldrich, A5486, Milwaukee, WI, USA) was administered to 40 mice by subcutaneous injection. From the second week, mice were given water with 2% Dextran sulfate sodium (DSS, molecular weight 36,000–50,000, MP Biochemicals, 0210151680, Santa Ana, CA, USA) every two weeks for 3 times. Mice were then divided into four groups (n = 10): AOM/DSS model, AOM/DSS + AKBA (30 mg/kg), AOM/DSS + AKBA (90 mg/kg) groups and 5-Fluorouracil (5-Fu, 20 mg/kg, Solarbio, IF0170) groups. For the control group and AOM/DSS model group, mice received sterile distilled water drinking. For the AOM/DSS + AKBA groups, mice were received the corresponding dose of AKBA by intragastric administration for 7 weeks. The 5-Fu group received intraperitoneal 5-Fu injections weekly. The mice’s weight was measured every week, and mouse activity status, fecal traits, as well as hematochezia were monitored and measured daily, with mice being sacrificed directly on the last day without drug administration. At the end of the experiment, all the mice were euthanized and the tumor number of colon, as well as the tumor volume (mm^3^, major diameter × minor diameter^2^/2) were recorded. The colon tissues were subsequently collected for further study.

### 2.15 Histological Analysis and Intestinal Tissue Hematoxylin-Eosin (H&E) Staining

Colon samples from mice were fixed in 4% paraformaldehyde, embedded in paraffin wax, and cut into 4 μm transverse sections, then stained with H & E as described in previous research [24]. Images were taken with a light microscope (Ti-U Nikon eclipse, Tokyo, Japan).

### 2.16 Meso Scale Discovery (MSD) Assay

Inflammation factors were significantly associated with colon cancer [25]. The mice were anesthetized with sodium pentobarbital, and blood was collected from the fundus venous plexus, centrifuged at 1000 rpm using a centrifuge (Allegra X-22R, Beckman coulter, Brea, CA, USA) for 10 min to obtain the serum. spleens were homogenized in PBS (1:10) and centrifuged at 2000 rpm for 10 min. Supernatant protein concentration was measured by BCA assay, and the samples were tested by V-PLEX Proinflammatory Panel 1 Mouse Kit (Univ Life Science and Technology Co., Ltd., No. K15048D-1, Shanghai, China) using MSD hypersensitive factor electrochemical luminescence analyzer (Meso scale disc., SQ120, Rockville, MD, USA).

### 2.17 Immunohistochemistry

The colonic tissue was fixed with 4% formaldehyde (Servicebio Life Science and Technology Co., Ltd., G1101—500 mL, Wuhan, China) embedded in paraffin, and sectioned. Slides were deparaffinized, rehydrated, and washed in PBS. Antigen retrieval was done with citrate buffer (Solarbio, G2700) at 100°C for 10 min slides were treated with 3% H_2_O_2_ to block endogenous peroxidase and blocked with 5% BSA (Solarbio, A8020) for 30 min. Primary anti-Ki67 antibody (1:500, Abcam, ab15580) was applied overnight, followed by 1 h incubation with biotinylated Goat Anti-Rabbit IgG (1:200, Cell Signaling Technology, 35401). Staining was completed with diaminobenzidine (DAB Substrate kit, Solarbio, SA1010), and hematoxylin (Solarbio, G1120). A microscope (Ti-U Nikon Eclipse, Tokyo, Japan) was used to take photographs of stained sections.

### 2.18 Gut Microbiota Profiling and Analysis

The profiling and analysis of gut microbiota in fecal samples from CRC mice, both treated and untreated with AKBA, were conducted using metagenomic sequencing and analysis methods as previously described [26,27]. The alpha and beta diversity were calculated in Qiimeplatform (http://qiime.org/scripts/assign_taxonomy.html (accessed on 10 January 2025) and RDP Classifier (version 2.11, http://sourceforge.net/projects/rdp-classifier/ (accessed on 10 January 2025). Community differences were analyzed using permutational multivariate analyses of variance (PERMANOVA) with 1000 iterations employing the Arrhenius z distance [28].

### 2.19 Statistical Analysis

Data are expressed as means ± SEM (the standard error of the mean), obtained from three separate experiments. GraphPad Prism 8.0 (GraphPad Software, San Diego, CA, USA) was utilized for creating graphs and conducting statistical analyses. For comparison between the two groups, unpaired Student’s *t*-tests were used. For multiple comparisons, one-way ANOVA followed by Bonferroni *post-hoc* tests was conducted. A *p*-value of less than 0.05 was considered statistically significant.

## 3 Results

### 3.1 AKBA Inhibited Proliferation, Arrested Cell Cycle, and Induced Apoptosis of Human CRC Cells

**Figure 1:**
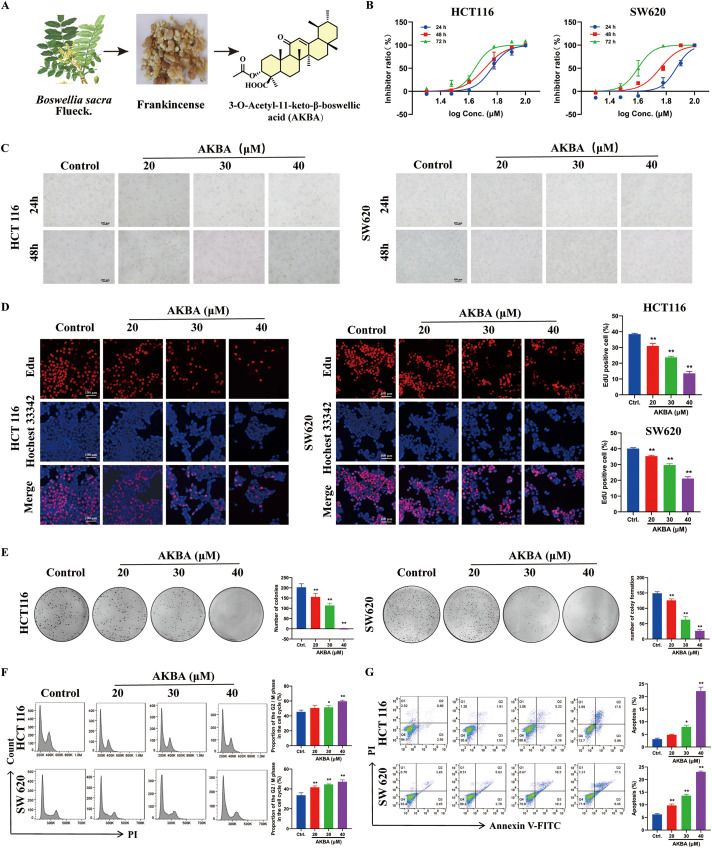
AKBA suppresses the proliferation of colorectal cancer (CRC) cells. (**A**) Image of *Boswellia carterii* Birdw, the image of resin seeping from the bark, and the chemical structure of 3-O-Acetyl-11-keto-β-boswellic acid (AKBA); (**B**) Cell proliferation assay was conducted on HCT116 and SW620 cells treated with different concentrations of AKBA at 24, 48, and 72 h were detected by Cell Counting Kit-8 (CCK-8 assay); (**C**) The morphology of HCT116 and SW620 cells treated with AKBA at 20, 30, and 40 μM. Scale bar = 100 μm; (**D**) DNA synthesis was assessed using 5-ethynyl-2^′^-deoxyuridine (EdU)-DNA assay after treatment with 0, 20, 30, and 40 μM AKBA for 24 h. Scale bar = 100 μm; (**E**) The ability of colony formation was checked after treatment of 0, 20, 30, and 40 μM AKBA; (**F**) Propidium iodide (PI) staining was used to analyze the distribution of cell cycle phases after treatment of 0, 20, 30, and 40 μM AKBA for 24 h by Flow cytometry; (**G**) Annexin V-FITC/PI staining was used to determine the apoptosis rate after treatment of 0, 20, 30, and 40 μm AKBA for 24 h by Flow cytometry. Note: *p < 0.05, **p < 0.01, compared with the control group

To check if AKBA has an inhibitory effect on the growth of CRC, CCK-8 assay was carried out. The Boswellia carterii Birdw, resin seeping from the bark, and the chemical structure of AKBA was depicted in Fig. 1A. Our results revealed that AKBA significantly reduced the growth of HCT116 and SW620 cells in a time- and dose-dependent manner. The IC_50_ values for AKBA in HCT116 cells at 24, 48, and 72 h were 41.86, 20.2, and 15.02 μM; and the values for SW620 were 74.2, 57.3, and 39.8 μM at 24, 48, and 72 h, respectively (Fig. 1B).

Therefore, AKBA at concentrations of 20, 30, and 40 μM were used to further investigate its anti-CRC effects and the underlying mechanisms. The morphology with HCT116 and SW620 cells was changed after treated by AKBA for 24 and 48 h (Fig. 1C). To check the inhibitory effects of AKBA on cell proliferation, an EdU-DNA synthesis assay was also done. After 24 h of treatment with AKBA, a dose-dependent decrease in the proliferation of HCT116 and SW620 cells was observed (Fig. 1D). Furthermore, the rate of clone formation of HCT 116 and SW620 were significantly decreased as concentrations of AKBA was increased (Fig. 1E).

To check whether AKBA affects the cell cycle and apoptosis of HCT116 and SW620 cells, Flow cytometry was used. Results indicated that AKBA treatment arrested the cell cycle at the G2/M phase (Fig. 1F) and increased apoptosis rates in a dose-dependent manner (Fig. 1G). Taken together, these findings suggest that AKBA exerts a growth-inhibitory effect on CRC cells.

### 3.2 AKBA Differential Gene Expression and Enrichment Analysis in HCT116 and SW620 Cells Which Were Treated with AKBA

**Figure 2:**
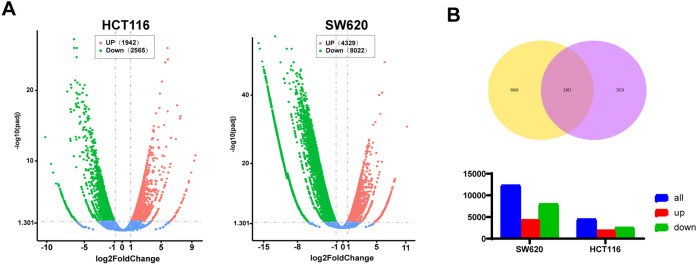

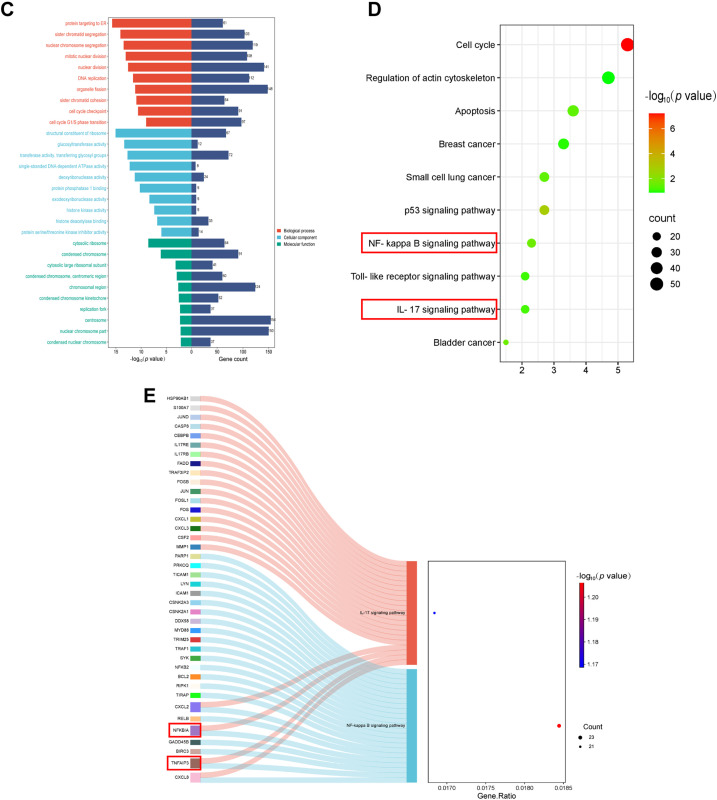
AKBA influences signaling pathways related to inflammation. (**A**) Volcano plot showing the differentially expressed genes in HCT116 and SW620 cells after 48 h of AKBA treatment; (**B**) Venn diagrams illustrating the differentially expressed genes in HCT-116 and SW620 cells; (**C**) Gene Ontology (GO) enrichment analysis for molecular functions (MF), biological processes (BP), cellular components (CC), and molecular functions (MF) of the differentially expressed genes; (**D**) Kyoto Encyclopedia of Genes and Genomes (KEGG) pathway enrichment analysis of the differentially expressed genes; (**E**) Sankey showed differentially expressed genes in the NF-κB pathway and IL-17 pathway

To further explore the anti-tumor mechanism of AKBA, an RNA-seq assay was carried out. Compared to control cells, |log_2_(Fold Change)| ≥ 3 and adjust *p* value ≤ 0.05 (Fig. 2A), a total of 2483 differential expression genes altered in both HCT116 and SW620 cells (Fig. 2B). Gene ontology enrichment analyses showed that the enriched biological processes involved cell cycle checkpoint, DNA replication, protein targeting to ER, sister chromatid segregation and G1/S phase transition. The identified cellular components were associated with a structural constituent of ribosome, glucosyltransferase activity, single-stranded DNA-dependent ATPase activity, protein phosphatase 1 binding, and histone kinase activity. Molecular functions included cytosolic ribosome, condensed chromosome, replication fork, centrosome, nuclear chromosome part, and condensed nuclear chromosome (Fig. 2C). The Kyoto Encyclopedia of Genes and Genomes (KEGG) pathway enrichment analysis showed significant changes in several biological processes including cellular senescence, cell cycle, DNA replication, NF-κB pathway, p53 signaling pathway, apoptosis and IL-17 pathway (Fig. 2D). A complete list of top 20 KEGG pathways related to cell growth, inflammation and tumors were listed in Table S3. The identified enriched KEGG pathways, including p53 signaling pathway, IL-17 signaling pathway, MAPK signaling pathway and NF-κB pathway, have been implicated in inflammation and tumor progression of CRC. Notably, the NF-κB signaling pathway is closely associated with both tumor development and inflammatory processes [29]. Additionally, cytokines are also associated with the NF-κB signaling pathway, thereby contributing to inflammation and cancer. Hut genes of the NF-κB pathway and IL-17 pathway were illustrated in Fig. 2E. All in all, these results indicated that the mechanism of AKBA was closely associated with the NF-κB pathway.

### 3.3 AKBA Suppresses the NF-κB Pathway in Colon Cancer Cells

**Figure 3:**
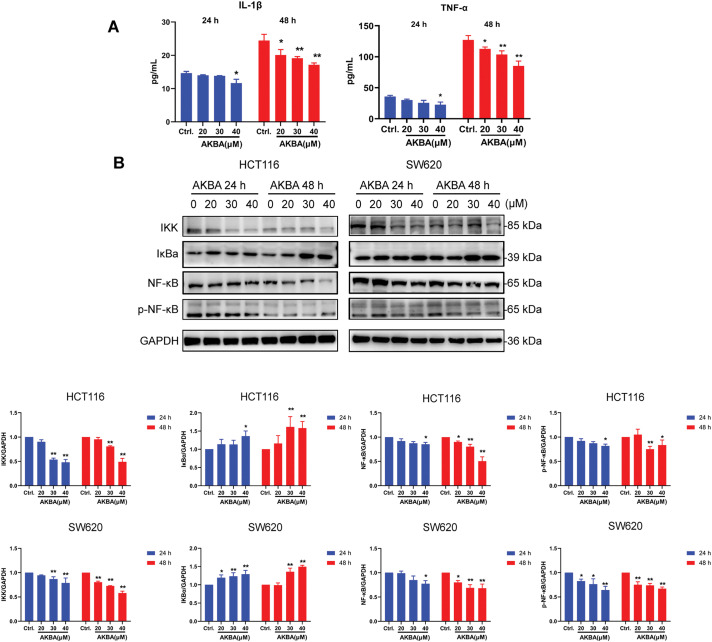

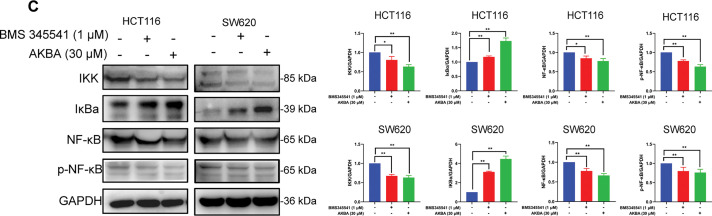
AKBA suppresses the NF-κB signaling pathway in CRC cells. (**A**) The changes of IL-1β and TNF-α were confirmed by ELISA assay; (**B**) Expression of key proteins in the NF-κB signaling pathway was detected by Western blot after treatment with 0, 20, 30, and 40 μM AKBA for 24 and 48 h; (**C**) Expression of key proteins in NF-κB pathway was assessed by Western blot after treatment with 30 μM AKBA alone. Note: *p < 0.05, **p < 0.01, compared with the control group

It was well known that alteration in the expression of various inflammatory factors was related to the NF-κB pathway during colon cancer. To confirm the effects of AKBA on inflammatory factors and the NF-κB pathway, ELISA analysis was carried out to check the concentrations of cytokines in the cell supernatant, as well as Western blot was checked the total and phosphorylated (*p*) levels of NF-κB, NF-κB, IκB, and IKK in HCT116 and SW620 cells treated by AKBA. Results from ELISA analysis showed that AKBA significantly reduced the levels of TNF-α and IL-1β compared to the untreated control group at 48 h (Fig. 3A). Furthermore, results from Western blot showed that the protein expression of NF-κB, p-NF-κB and IKK were obviously decreased whereas the expression of IκB was increased after treatment of AKBA (Fig. 3B). This means that AKBA can inhibit the activation of the NF-κB pathway. To further confirm that the NF-κB pathway was involved in the effect of AKBA on the CRC. BMS345541 (an IKK inhibitor) was also used to treat HCT116 and SW620 cells. It was showed in Fig. 3C that BMS345541 reduced the expression of IKK, NF-κB, and p-NF-κB whereas expression of IκB was increased in cells treated by BMS345541, which is similar to AKBA. Collectively, these findings suggest that AKBA exerts its inhibitory effects on CRC by regulating NF-κB pathway.

### 3.4 AKBA Inhibited the Levels of Inflammatory Factor Both in LPS-Induced RAW 264.7 Cell and in the LPS-Induced Mice with Inflammation

**Figure 4:**
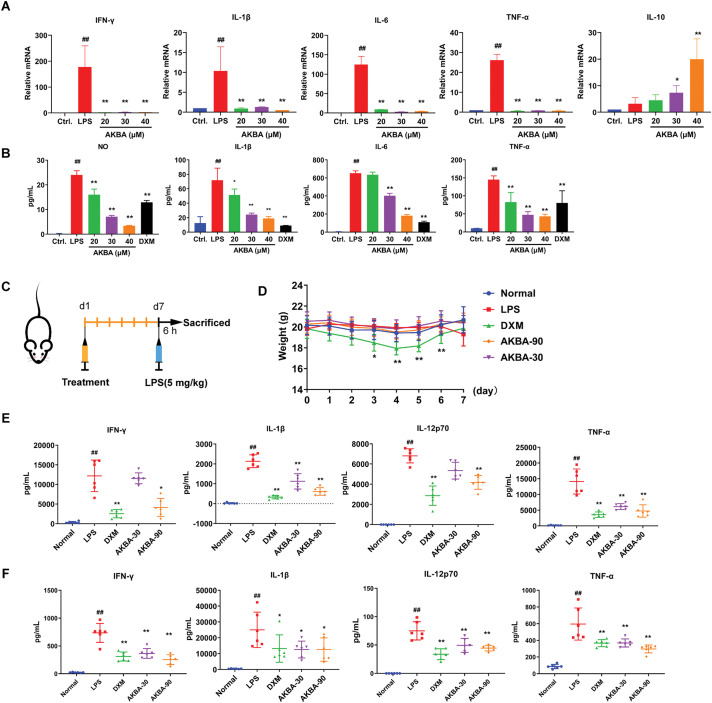
AKBA inhibits inflammation in both LPS-induced RAW 264.7 cell and LPS-induced mice with inflammation. (**A**) The mRNA levels of Interleukin-1β (IL-1β), Interleukin-6 (IL-6), Interferon-γ (IFN-γ), tumor necrosis factor-α (TNF-α), and Interleukin-10 (IL-10) in lipopolysaccharide (LPS) induced RAW 264.7 cell were measured by real-time quantitative PCR (RT-qPCR); (**B**) Concentrations of Nitric Oxide (NO), IL-6, IL-1β, IL-6, and TNF-α in LPS-treated RAW 264.7 cells were assessed by enzyme-linked immunosorbent assay (ELISA); (**C**) Schematic of LPS-induced inflammation model used to study the anti-inflammatory effects of AKBA; (**D**) Changes in body weight throughout the duration of the experiment; (**E**) Levels of IL-1β, IFN-γ, Interleukin-12p70 (IL-12p70), and TNF-α in the serum of each group (n = 6) were detected by MSD analysis; (**F**) Levels of IL-1β, IFN-γ, IL-12p70, and TNF-α in the spleen of each group (n = 6) were detected by MSD analysis. Note: ^##^*p* < 0.01, compared with the normal group; *p < 0.05, **p < 0.01, compared with the LPS group

To further investigate the effects of AKBA on the inflammatory factors both in cellular and animal models of inflammation, we measured the levels of inflammatory factors in RAW 264.7 cells, as well as in the serum and spleen of the mice. In the LPS-induced RAW 264.7 cell model, LPS treatment significantly increased the mRNA levels of IL-6, IL-1β, IFN-γ, TNF-α and IL-10. AKBA treatment, however, reduced the mRNA levels of IL-6, IL-1β, IFN-γ, and TNF-α, while increasing the level of IL-10 compared to the LPS-treated group (Fig. 4A). In addition, results from the ELISA assay showed that AKBA decreased the levels of NO, IL-1β, IL-6 and TNF-α in LPS-induced RAW264.7 cells (Fig. 4B). Next, we used LPS-induced inflammation mice model to further confirm the effects of AKBA on cytokines. A schematic diagram of mice treatment is illustrated in Fig. 4C. The body weight of the DXM group decreased following DXM administration, with significant weight loss observed on days 3,4,5, and 6 compared to the normal group. In contrast, AKBA had no effect on the body weight of the mice (Fig. 4D). The treatment of LPS led to a notable increase of inflammatory factors, such as IL-1β, IFN-γ, IL-12p70 and TNF-α both in serum and spleen compared to the control group. After treatment of AKBA, the levels of IL-1β, IFN-γ, IL-12p70 and TNF-α were decreased both in serum and spleen (Fig. 4E,F). AKBA also decreased the level of IL-5 in serum (Fig. S4A), as well as IL-2 and IL-6 in the spleen (Fig. S4B), which indicated that AKBA alleviated systemic inflammation. These findings indicate that AKBA possesses significant anti-inflammatory properties, as shown in both *in vitro* and *in vivo* models.

### 3.5 AKBA Suppressed the Tumorigenicity and Ameliorated AOM/DSS-Induced Colonic Pathological Changes in Mice

**Figure 5:**
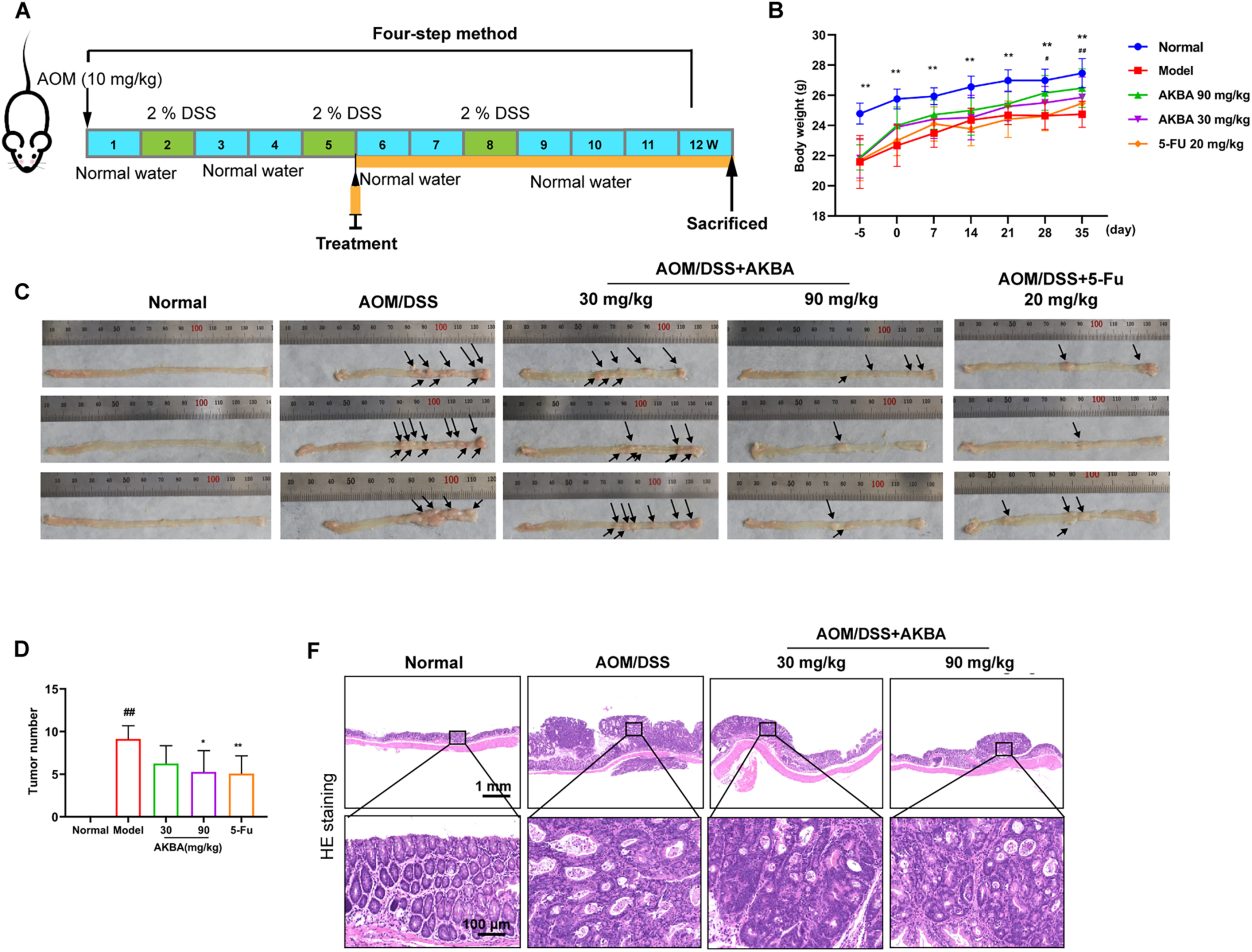

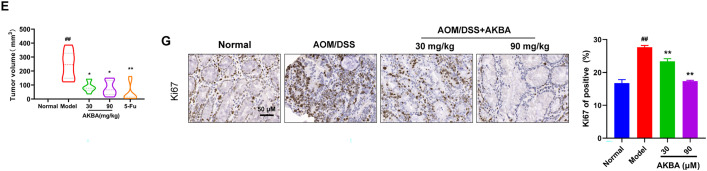
AKBA reduces colorectal tumorigenesis in mice. (**A**) Schematic illustration of AOM/DSS induced colitis-associated CRC model used to evaluate the anti-tumor effects of AKBA; (**B**) Representative images of colon tumors from normal control, vehicle, and various treatment groups; (**C**) Changes in body weight throughout the AKBA treatment period, the black arrows indicated the tumor formed on the colon tissue; (**D**) Tumor numbers of the mice at the end of the experiment; (**E**) Tumor volumes of the mice at the end of the experiment; (**F**) Representative H&E staining images of colonic tissue from normal control, vehicle, and AKBA-treated groups; (**G**) Representative images of colonic tissue from normal control, vehicle, and AKBA-treated groups. Note: ^#^*p* < 0.05, ^##^*p* < 0.01, compared with the normal control group; *p < 0.05, **p < 0.01, compared with the AOM/DSS group

Previous study had indicated that the NF-κB pathway is significantly activated in DSS-induced inflammatory bowel disease [12]. To check whether AKBA suppressed the tumorigenicity of CRC *in vivo*. A CRC model induced by AOM/DSS in BalB/c mice was established, and the mice were treated with AKBA at doses of 50 and 100 mg/kg daily for a duration of 7 weeks (Fig. 5A). During the 7 weeks’ administration of AKBA, no abnormal behavior or obvious weight loss was observed (Fig. 5B). The representative images of colon tumors from normal control, vehicle and AKBA treatment group were shown in Fig. 5C. Oral administration of AKBA significantly reduced the number (Fig. 5D) and the volume of tumors (Fig. 5E). HE staining of colon tissue showed that AKBA significantly alleviated the pathological changes of colon cancer tissue in mice (Fig. 5F). Immunohistochemistry of intestinal tissue showed that the expression of Ki67 was reduced in the in AKBA-treated group compared to the vehicle group (Fig. 5G). The index of the thymus, spleen, and colon wasn’t significantly changed after treatment of AKBA compared to the normal group (Fig. S5A–C). Together, all these results showed that AKBA suppressed the tumorigenicity and ameliorated AOM/DSS-induced colonic pathological changes in mice.

### 3.6 AKBA Restored the Disordered Intestinal Gut Microbiota

**Figure 6:**
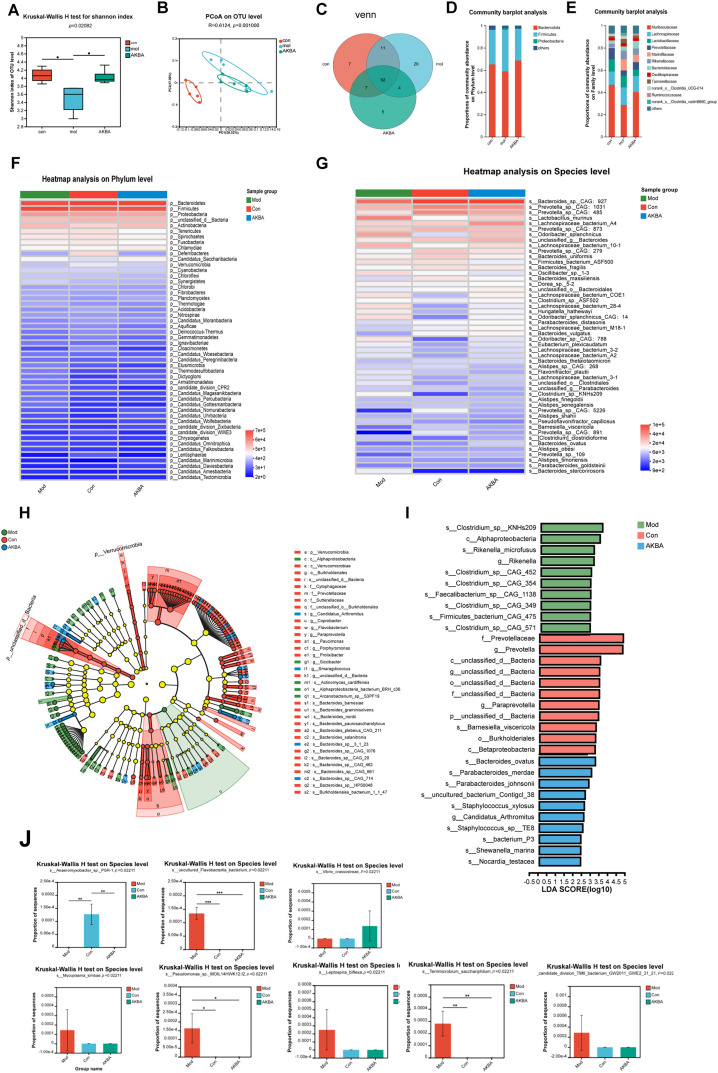
AKBA corrects the abnormal alterations in gut microbiota. (**A**) Shannon index at the operational taxonomic units (OTUs) level for normal control, vehicle, and AKBA-treated groups; (**B**) Principal coordinate analysis (PCoA) plot of OTUs for normal control, vehicle, and AKBA treated groups; (**C**) The Venn diagram illustrates the overlap of shared species among the different groups; Relative abundance of intestinal flora in the normal group, AOM/DSS induced CRC model group, and AKBA-treated group at phylum levels (**D**) and family levels (**E**); (**F**) Heatmap showing the hierarchical clustering distance of samples; (**G**) Analysis of species differences; (**H**) LEfSe multi-level species difference discriminant analysis; (**I**) Biomarkers of the differential change in each group; (**J**) Differentially altered flora significantly affected by treatment of AKBA. Note: *p < 0.05, **p < 0.01, ***p < 0.001, compared with the AOM/DSS group

The role of microbial communities in human pathophysiology, especially in oncogenesis, has become a focal point of contemporary biomedical research. Gastrointestinal cancers demonstrate particularly significant interactions with enteric microbiota, a relationship attributed to their anatomical adjacency within the digestive ecosystem. This unique biological positioning has rendered gut microbiome-oncology correlations a priority subject in current translational research, with numerous studies elucidating microbial contributions to carcinogenesis, tumor progression, and therapeutic responses [30]. To determine whether AKBA influences the gut microbiota in mice with CRC, the intestinal flora of the mice was analyzed by the 16S rDNA pyrosequencing. After the amplification of 338F_806R, a total of 930653472 (bp) bases were detected, optimized for 649635838 OTUs, with an average length of 420.22. A total of 575 species were identified. When compared with the control group, significant differences were observed in the Shanno index among the control, model, and AKBA groups (Fig. 6A and Table S4). Additionally, differences among the groups were also significant in the PCoA analysis (Fig. 6B). The 92 differential species were shared among the 3 groups (Fig. 6C). There was no significant difference in dominant species at the phylum level (Fig. 6D). At the family level, the dominant species included Muribaculaceae, Lachnospiraceae, Marinifilaceae, and Rikenellaceae, Bacteroidaceae (Fig. 6E). The top 20 species with the most pronounced changes at both the phylum and species level were shown in Fig. 6F,G. LEfSe multi-level species difference discriminant analysis (which includes various levels including phyla, class, genus, family, order and species) was conducted to examine the hierarchical differences among species. The LDA value was used to measure the differential effect of species, suggesting that the species may play an important role in the process of environmental change. This analysis can also be used as one of the ways to find the biomarker between the control group and the treatment groups (Fig. 6H,I). The intestinal microflora consists of directly carcinogenic microflora, bacterial toxins (e.g., colibactin, BFT), passenger bacteria (opportunistic microorganisms), Fungi (*Penicillium* spp., *Aspergillus* spp., and *Fusarium* spp.). Changes in the microbiota can lead to dysbiosis and the development of CRC [31]. Compared with model group, *Clostridium* sp. KNHs209, *Bacteroides ovatus* [32], *Bacteroides* sp. CAG:20, *Parabacteroides merdae*, *Paraprevotella clara*, *Rikenella microfusus*, *Bacteroides* sp. CAG:702 were increased. Whereas *Barnesiella viscericola* [33], *Bacteroides oleiciplenus*, *Coprobacter secundus* [34], *Bacteroides* sp. CAG:633, *Bacteroides* sp. CAG:462, *Bacteroides salyersiae*, *Bacteroides salanitronis* were decreased after treatment of AKBA (Fig. 6J). All these results showed that AKBA can restore the intestinal microbiota by increasing probiotics and reducing harmful bacteria.

**Figure 7:**
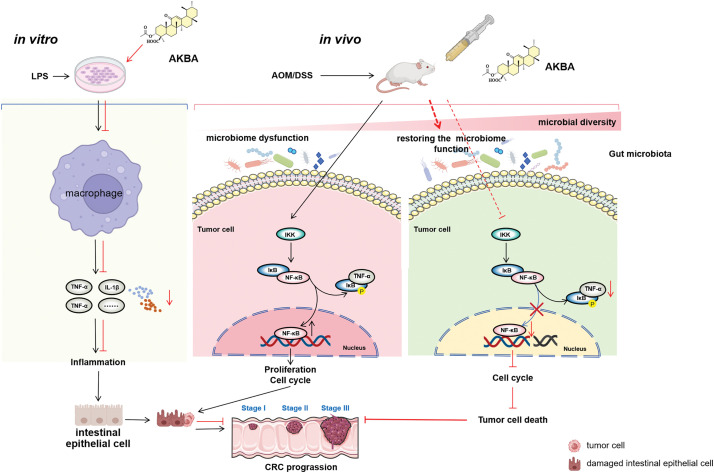
Proposed molecular mechanisms underlying the anti-colon cancer effects of AKBA. AKBA was shown to inhibit CRC progression through mechanisms including the alleviation of inflammation, reducing the activity of the NF-κB signaling pathway, and restoration of gut microbiota dysbiosis. The Figure was created with BioRender.com

Based on the above findings, we have proposed a mechanistic scheme, shown in Fig. 7, demonstrating that AKBA exerts an anti-cancer effect on the colon by modulating inflammation and the gut microbiota. AKBA inhibits the production of pro-inflammatory cytokines, such as IL-1β and TNF-α in macrophages, thus reducing systemic inflammation. AKBA also inhibits tumor cells by reducing the NF-κB signaling pathway, which reduces TNF-α production and prevents further inflammation. In addition, AKBA improves microbial diversity and increases beneficial gut bacteria that further support anti-inflammatory and anti-tumor activities. Taken together, these effects help AKBA to suppress the progression of colon cancer.

## 4 Discussion

Colon cancer is the most common cancer all over the world. Despite the availability of various treatment methods, it continues to have a poor prognosis. It is an urgent need to find new therapeutic drugs. In this study, we found that AKBA inhibited the proliferation and colony formation of the CRC cells, induced apoptosis, and arrested the cell cycle at the G2/M phase. The mechanisms of AKBA were related to decreased inflammation and the down-regulation of NF-κB signaling pathway. In addition, AKBA also suppressed the colitis-associated tumorigenesis of CRC in AOM/DSS-induced mice and restored gut microbiota dysbiosis. These findings demonstrated that AKBA could be a promising drug for treating inflammation-related CRC.

Boswellic acids exhibit a significant anti-proliferative and anti-cancer effects. AKBA has consistently demonstrated the ability to inhibit the proliferation of various cancer cell lines in a time- and dose-dependent manner [35,36]. AKBA can arrest the cell cycle at the G2/M phase in glioblastoma cells [9]. AKBA demonstrates potent anti-cancer effects via multiple mechanisms. These mechanisms involve the induction of apoptosis, the arrest of cell cycle, and the inhibition of migration and invasion [37–39]. Consist with the previous study, we found that AKBA induced apoptosis, arrested the cell cycle at the G2/M phase, inhibited proliferation of human CRC cells.

Inflammation plays an important role in the development of colon cancer. Many colon cancers were caused by bowel inflammation, especially ulcerative colitis (UC) [3,12]. Cytokines along with the NF-κB pathway are pivotal regulators of inflammation process. Pro-inflammatory cytokines can trigger the NF-κB pathway and activated NF-κB can enhance production of cytokine, creating a positive feedback loop that worsens inflammation in various diseases including colon cancer [11,13]. Interleukin-6(IL-6) is a key cytokine that bridges chronic inflammation and CRC progression [40,41]. It plays a significant role in promoting tumor growth, metastasis, and immune evasion. Elevated IL-6 levels are often related to poor prognosis in CRC patients. IL-6, a key pro-inflammatory cytokine, has the ability to initiate the NF-κB pathway [42]. This activation, in sequence, modulates the expression of numerous genes related to inflammation, cell survival, and proliferation. Previous research has indicated that boswellic acid can effectively inhibit cytokine release, suppressing pro-inflammatory cytokines like TNF-α, IL-6, and IL-1β, which are critical to the inflammatory response [43]. Additionally, boswellic acids can disrupt the activation of the NF-κB pathway through the prevention of the phosphorylation and degradation of IκB proteins [44,45]. However, the effects of AKBA on colon cancer have not been investigated. In our study, we discovered that in a LPS-induced inflammatory model with RAW264.7 cells, AKBA remarkably decreased the release of inflammatory factors like IL-1β, IL-6, and TNF-α. AKBA also decreased cytokine production including TNF-α, IL-1a, IL-1β, IL-6, and IL-10 in both spleen and serum in a LPS-induced systemic inflammation model in mice. In addition, AKBA suppressed tumor progression in a mouse model of AOM/DSS-induced colitis-associated colon cancer. Additionally, our findings demonstrated that AKBA down-regulated the NF-κB pathway in colon cancer cells. Specifically, AKBA decreased the phosphorylation of NF-κB and IkB, indicating the inhibition of NF-κB pathway. Our findings align with previous research, confirming that AKBA can exert anti-inflammatory and anti-colon cancer effects by the modulating cytokines and the NF-κB signaling pathway.

The effect of AKBA on the NF-κB pathway is different from that of other anti-inflammatory agents. Traditional nonsteroidal anti-inflammatory drugs (NSAIDs), such as like ibuprofen and naproxen, mainly exert their effect by inhibiting the enzyme cyclooxygenase (COX), which reduces inflammation by lowering prostaglandin levels. In contrast, AKBA directly influences the NF-κB pathway by affecting important components such as IKK. Unlike NSAIDs, which can lead to gastrointestinal issues and potential dependence, AKBA appears to have a more selective effect on the NF-κB pathway without causing these negative side effects.

The gut microbiota is a community of microbes that live in the human gut, including bacteria, fungi, viruses, and other microorganisms. Intestinal flora interacts with colon epithelial cells and participates in important processes, such as digestion, absorption, metabolism, and immune response [4]. Bacteria can form tumor-coating biofilms that trigger NF-κB signaling which participates in the pro-inflammatory signaling cascades and upregulates the level of inflammatory mediator (IL-1 and IL-8). At the same time, microbial-derived bioactive compounds exhibit carcinogenic potential through induction of inflammatory mediators, notably TNF-α and IL-17, which establish a feed forward loop with dysregulated immune responses to drive oncogenic pathways. This molecular crosstalk fosters a chronic inflammatory microenvironment conducive to malignant transformation and metastatic progression. Changes in intestinal flora are closely related to the occurrence and development of CRC. The previous study showed that AKBA could modulate the gut microbiome composition of AKBA in healthy mice [46]. In this study, we found that AKBA can increase “good bacteria”/probiotics (such as *Clostridium* sp. KNHs209, *Bacteroides* sp. ovatus, *Parabacteroides merdae*, etc.) and reduce “bad bacteria”, harmful bacteria (such as *Barnesiella viscericola*, *Bacteroides oleiciplenus*, etc.). *Bacteroides ovatus* [31] had the potential to influence the efficacy of cancer therapy and was positively correlated with lung cancer. The reduction of *Clostridium* sp [47] was a potential target of excessive fructose intake associated with the rising prevalence of nonalcoholic fatty liver disease (NAFLD). At the same time, *Clostridium* sp. caused crosstalk with lithocholic acid (LCA) species aggravating the effect on ANFLD. *Barnesiella viscericola* and *Coprobacter secundus* were decreased after treatment of AKBA. *Barnesiella viscericola* was increased in the gut microbiome in polycystic ovarian syndrome (PCOS) patients with depression, but there have been no reports of a detailed study of the strain in tumors [34]. *Coprobacter secundus* [35] was found to have the highest discriminatory power in patients with metastatic and non-metastatic pancreatic cancer. Those probiotics were changed by treatment on AKBA, it is very interesting and will become the next study about AKBA on specific intestinal microorganisms.

AKBA has a wide range of pharmacological activities, including anti-inflammatory, anti-cancer, antimicrobial, and neuroprotective effects, highlighting its potential as a therapeutic drug for different diseases [42]. Conventional treatments for CRC, such as 5-fluorouracil (5-FU) and oxaliplatin, are associated with considerable side effects and may not be effective in all patients. In contrast, AKBA has shown significant anti-proliferative and anti-cancer properties in CRC cell lines and animal models. Furthermore, as a natural compound, AKBA is noted for its low toxicity and reduced incidence of adverse effects. However, the current study on AKBA’s efficacy in CRC treatment also has several limitations. Firstly, the use of animal models may not fully replicate the complexity of human CRC, limiting the generalizability of the findings. Exploring the use of patient-derived xenograft models could provide more accurate insights into AKBA’s therapeutic potential in human CRC. Secondly, the present study did not assess potential synergistic effects; thus, future research should examine the advantages of combining AKBA with other anti-cancer agents, including chemotherapeutic, targeted, and immunotherapeutic drugs. Lastly, the toxicity, pharmacokinetic properties, and dosage formulations of AKBA remain insufficiently understood. Further research is needed to fully explore these characteristics.

In conclusion, our study demonstrates that AKBA effectively arrests the cell cycle at the G2/M phase, induces apoptosis and inhibits the proliferation of CRC cells. Mechanistically, AKBA downregulates the NF-κB signaling pathway, decreases the levels of pro-inflammatory cytokines, such as TNF-α, IFN-γ, IL-1β, and IL-12p70, and reduces cellular inflammation. AKBA also exhibits anti-tumor effects in an AOM/DSS-induced mouse model of colitis-associated CRC and positively influences gut microbiota composition. These findings highlight AKBA’s potential as a novel therapeutic agent for inflammation-related CRC.

## Supplementary Materials













## Data Availability

The datasets generated and/or analyzed during the current study are available from the corresponding authors on reasonable request.

## Supplementary Materials

The supplementary material is available online at https://www.techscience.com/doi/10.32604/or.2025.062386/s1.
