# Supplementary figures and images for: 3-O-Acetyl-11-Keto-β-Boswellic Acid Suppresses Colitis-Associated Colorectal Cancer by Inhibiting the NF-Kb Signaling Pathway and Remodeling Gut Microbiota
Source: Oncol Res. 2025 Jul 18;33(8):1969–89. doi: 10.32604/or.2025.062386 (PMC12308244; doi:10.32604/or.2025.062386)

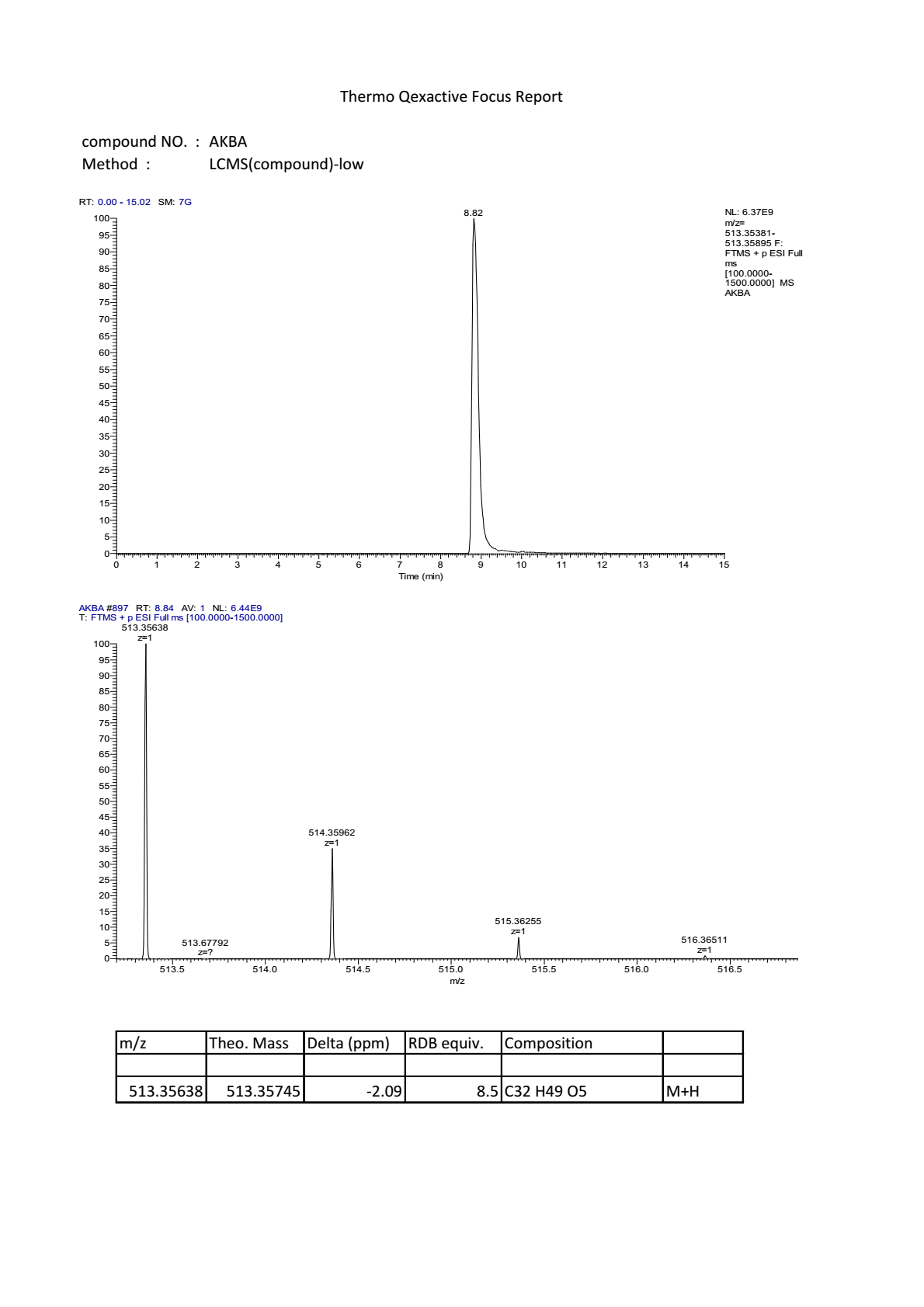

Supplement: Supplementary file 1 [file OncolRes-33-62386-s001.tif]

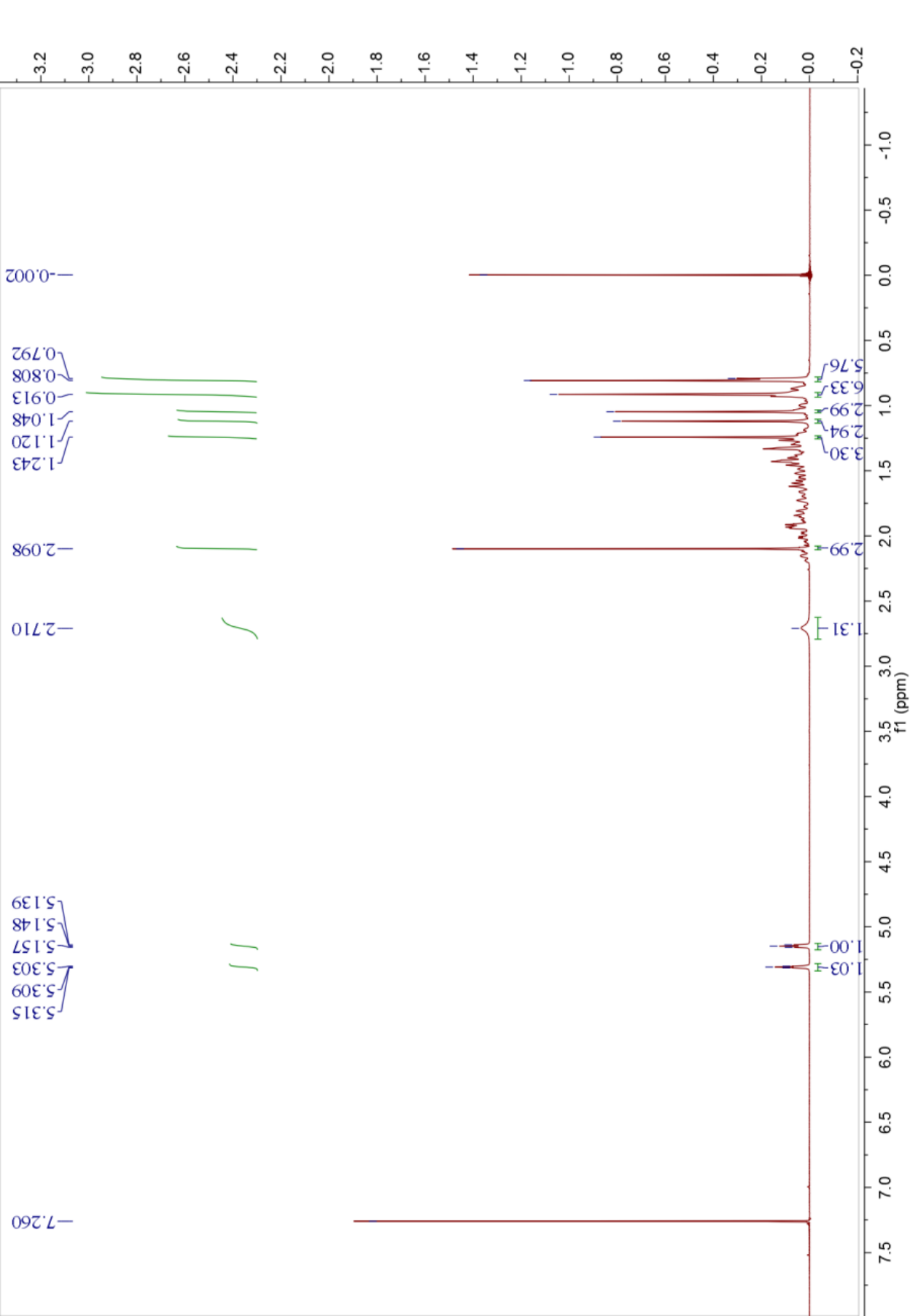

Supplement: Supplementary file 2 [file OncolRes-33-62386-s002.tif]

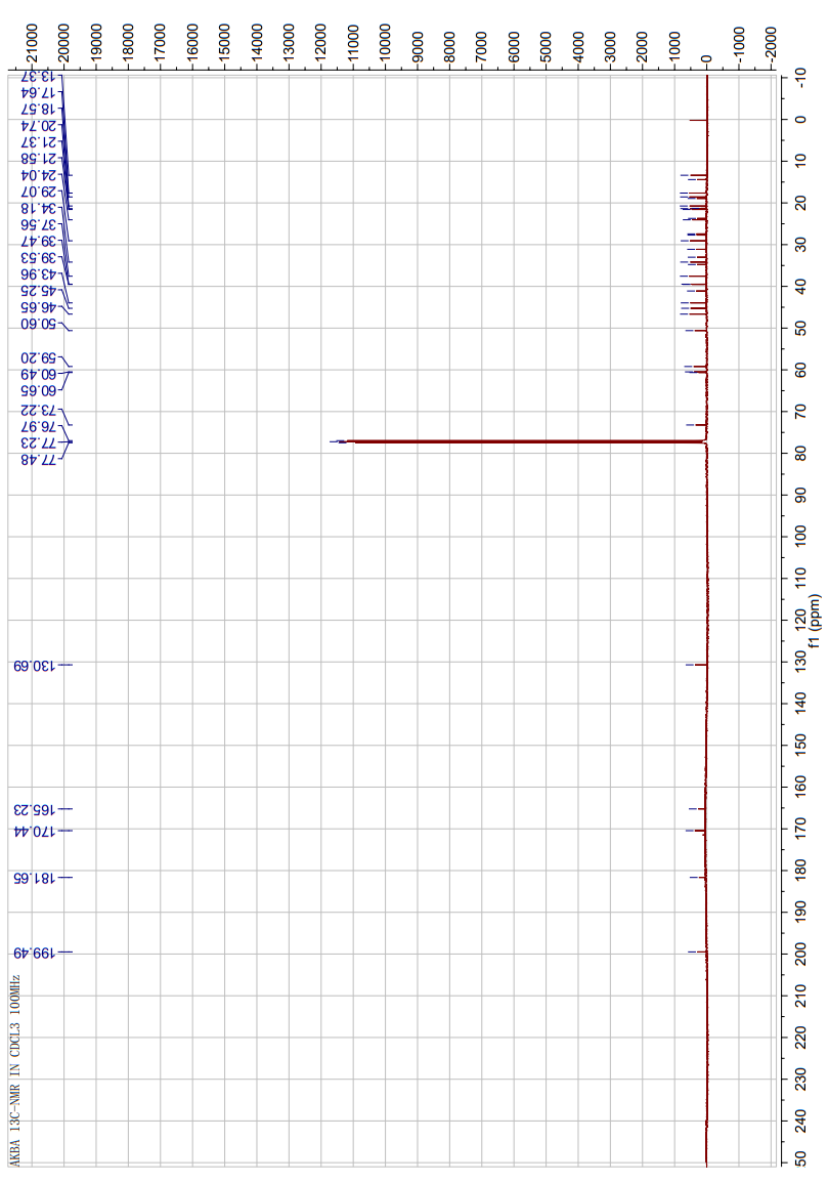

Supplement: Supplementary file 3 [file OncolRes-33-62386-s003.tif]

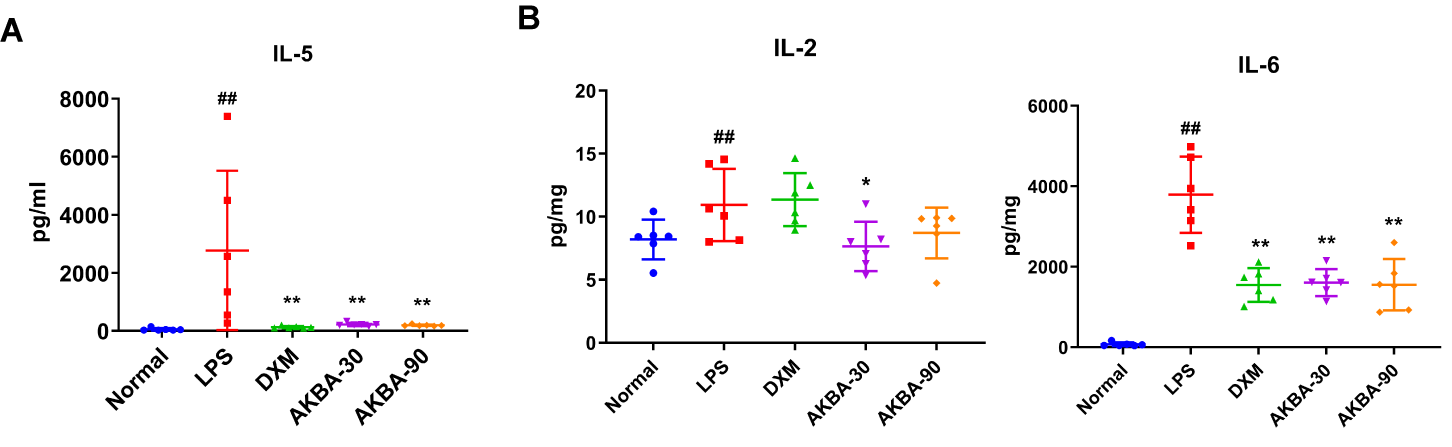

Supplement: Supplementary file 4 [file OncolRes-33-62386-s004.tif]

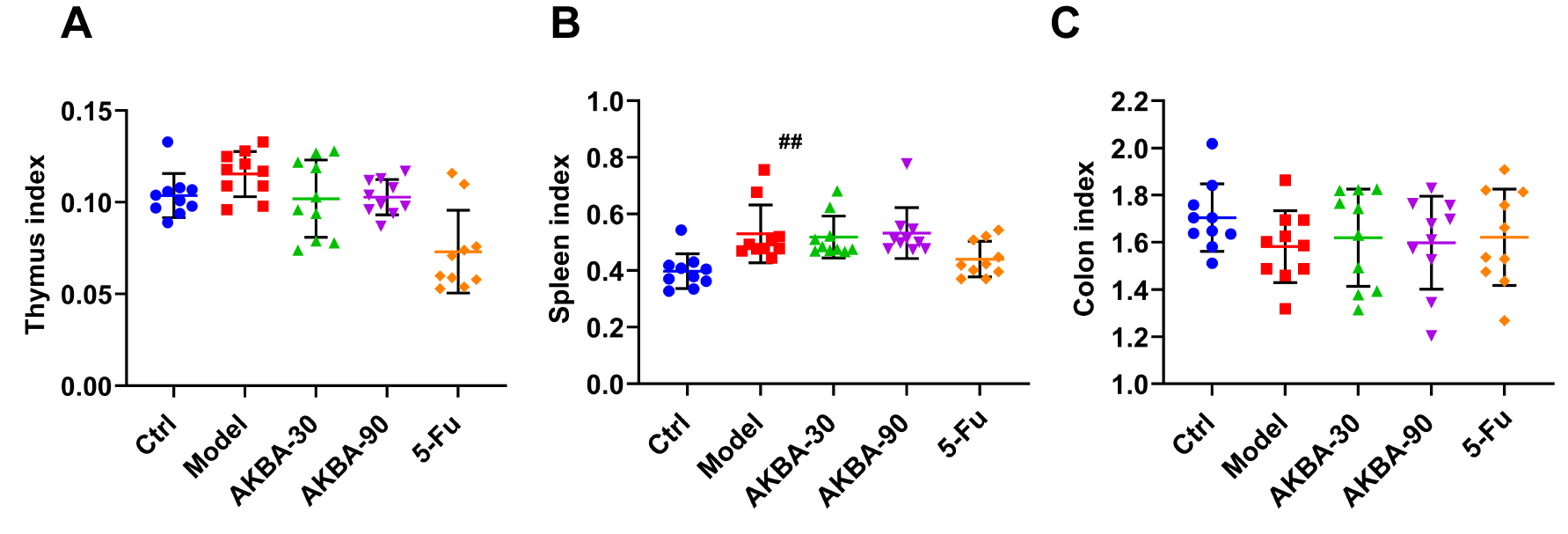

Supplement: Supplementary file 5 [file OncolRes-33-62386-s005.tif]
