# Supplementary material for: 3-O-Acetyl-11-Keto-β-Boswellic Acid Suppresses Colitis-Associated Colorectal Cancer by Inhibiting the NF-Kb Signaling Pathway and Remodeling Gut Microbiota
Source: Oncol Res. 2025 Jul 18;33(8):1969–89. doi: 10.32604/or.2025.062386 (PMC12308244; doi:10.32604/or.2025.062386)
Supplement: Supplementary file 6 [file OncolRes-33-62386-s006.docx]

**Supplemental material**

- 1. **Introduction of Chemotherapeutic drug**

AKBA was obtained as colorless needles; mp 268–270°C; and its molecular formula was determined as C_32_H_48_O_5_ on the basis of its HR-ESI-MS spectrum m/z 513.35638 [M+H]^+^ (calculated for C_32_H_49_O_5_: m/z 512.4) indicating nine indices of hydrogen deﬁciency (**Fig. S1**).

The ^1^H NMR spectrum showed eight methyl singlets and the ^13^C NMR spectrum revealed 32 carbon signals. Due to Ursane-type as pentacyclic triterpenoid isolated from the resin in previous studies,.^1^H NMR (400MHz CDCl_3_) *δ*_H_ 5.55(1H, s, H-12), 5.30(1H, brs, H-3), 2.08 (3H, s, CH_3_CO-), 1.23(3H, s, CH_3_-23), 1.14(3H, s, CH_3_-25), 1.19(3H, s, CH_3_-26), 1.34 (3H, s, CH_3_-27), 0.82(3H, s, CH_3_-28), 0.79(3H, d, *J* = 6.4Hz, CH_3_-29), 0.94(3H, s, CH_3_-30) in **Fig. S2**. ^13^C NMR (100MHz CDCl_3_) *δ*_C_ 34.6(C-1), 23.5(C-2), 73.0(C-3), 46.5(C-), 50.4 (C-5), 18.7(C-6), 32.8(C-7), 43.8(C-8), 63.3(C-9), 37.4(C-10), 199.2 (C-11), 130.5(C-12), 165.0(C-13), 45.1(C-14), 27.5(C-15), 27.2(C-16), 34.0(C-17), 59.0(C-18), 39.31(C-19), 39.27(C-20), 46.5(C-21), 40.9(C-22), 23.8(C-23), 181.8 (C-24), 13.2(C-25), 18.4(C-26), 21.1(C-27), 28.8(C-28), 17.4(C-29), 20.5(C-30), 21.33(CH_3_CO-), 170.2(CH_3_CO-) in **Fig. S3**.


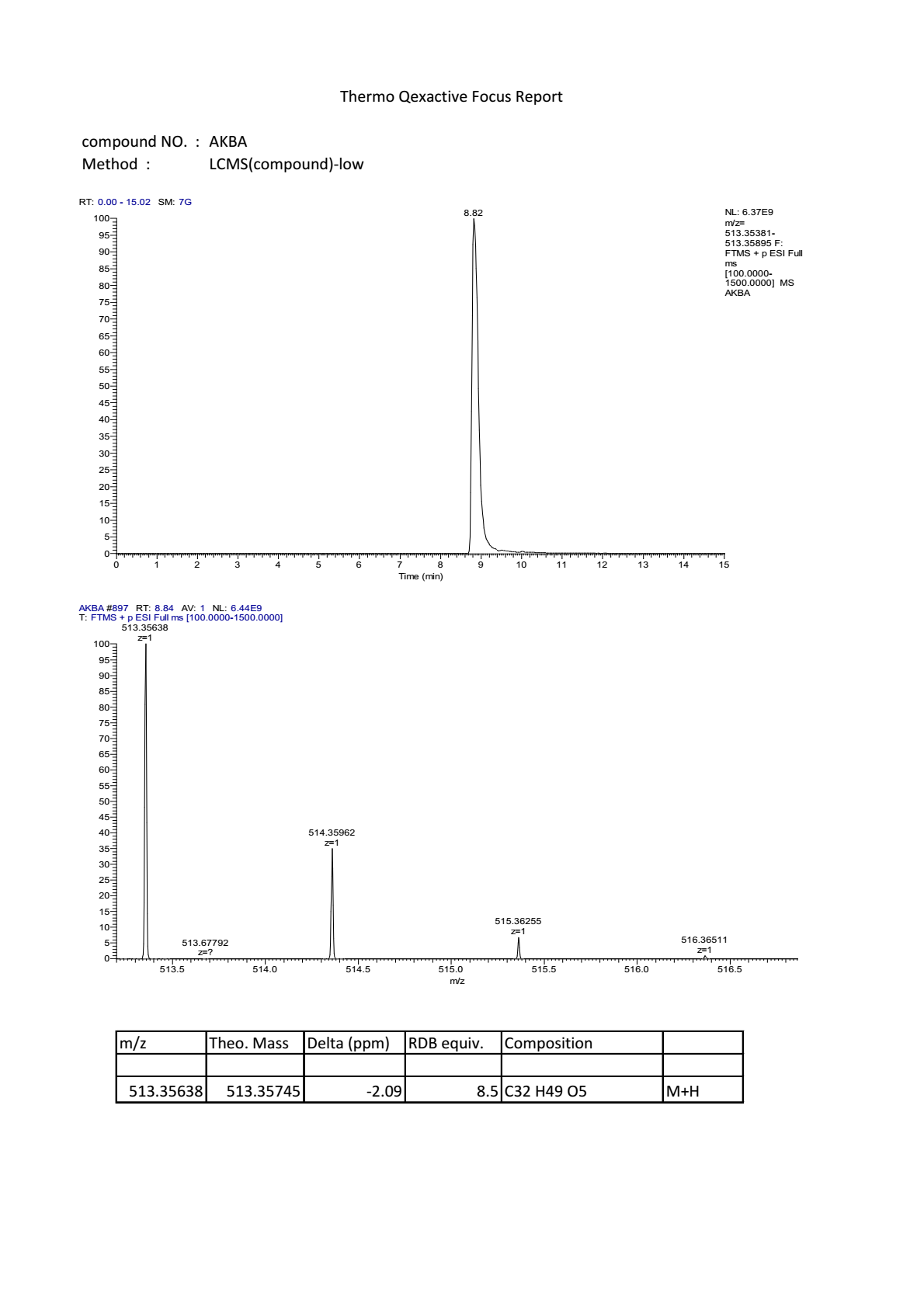


**Figure S1. HR-ESI-MS spectrum of AKBA.**


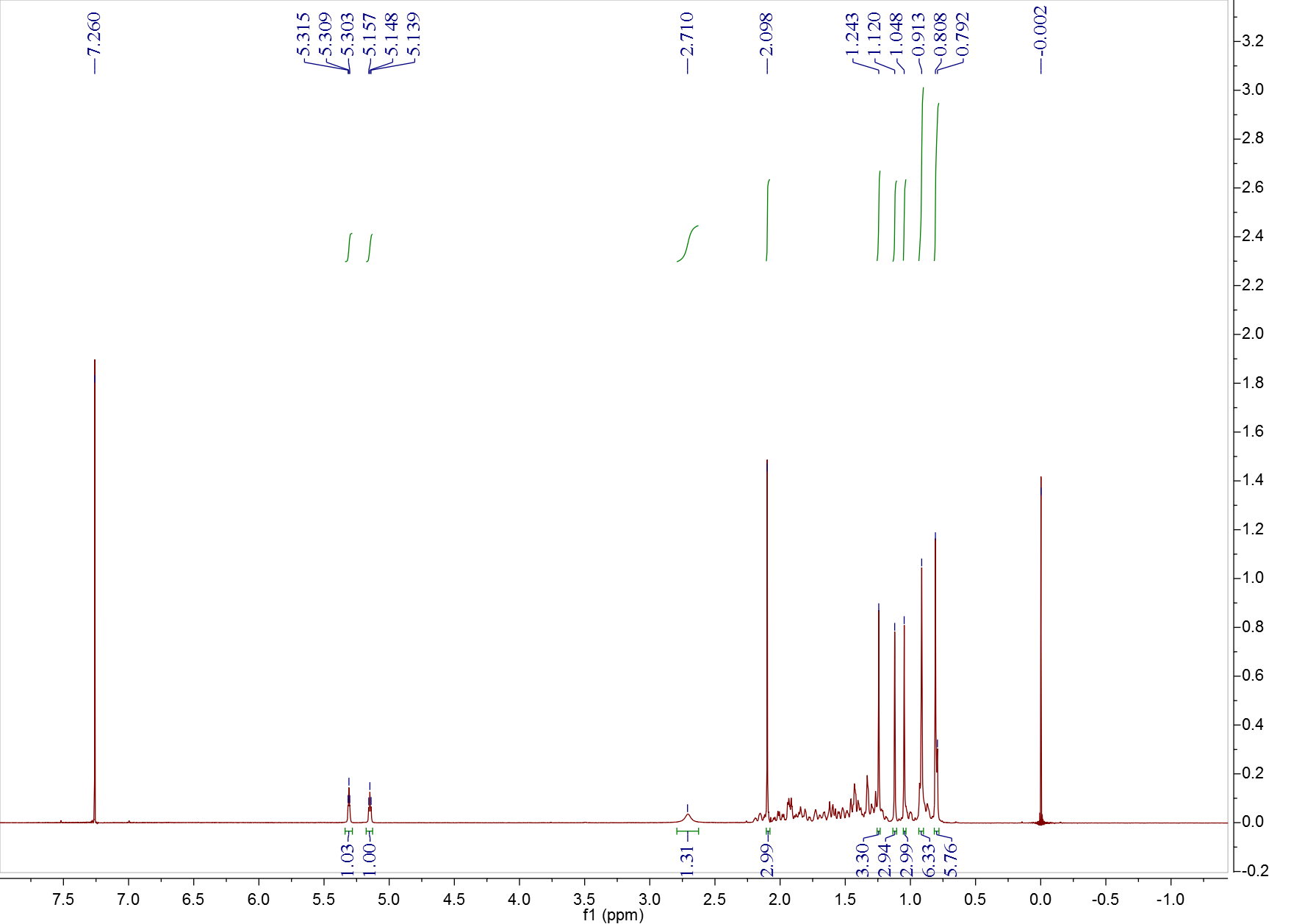


**Figure S2. The ^1^H NMR spectrum of AKBA (400 MHz, CDCl_3_).**


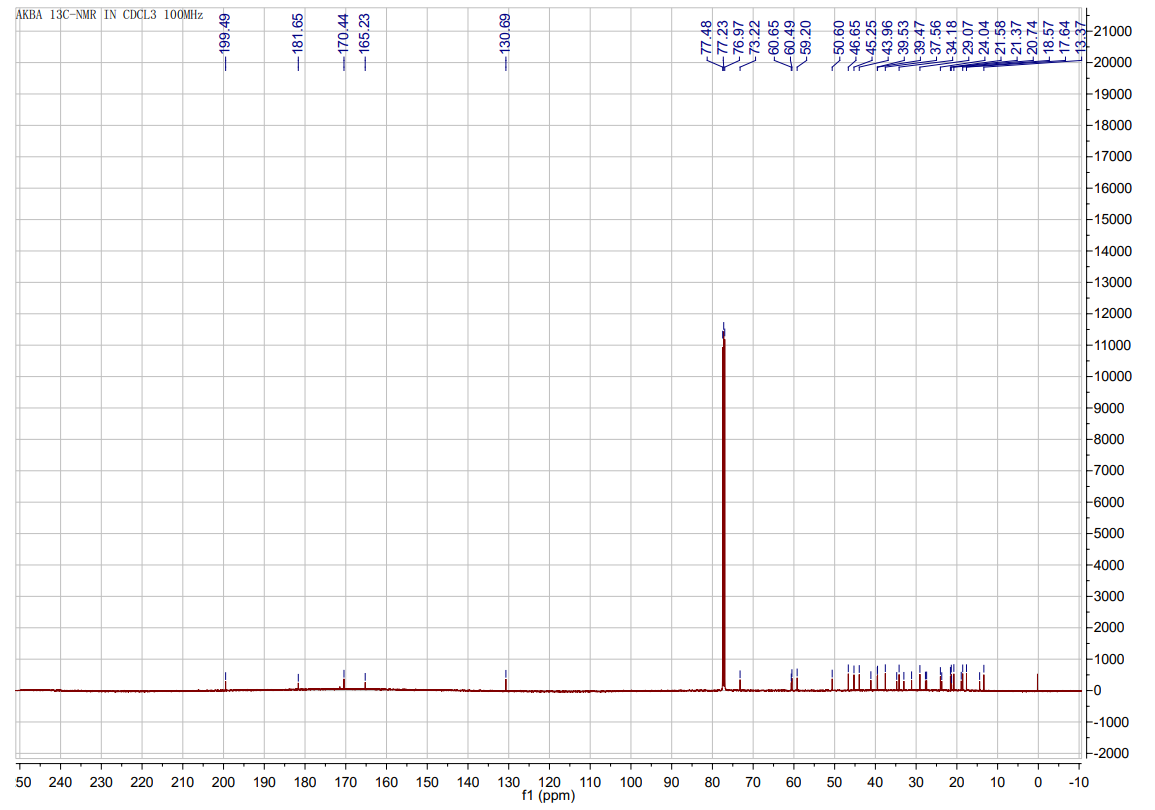


**Figure S3. The ^13^C NMR spectrum of AKBA (100 MHz , CDCl_3_).**

- 1. **The detailed descriptions of some of the methods**
     1. **Cell proliferation assay**

Cell Counting Kit-8 (CCK-8) assay was used to assess cell viability after exposure to AKBA for the indicated time points. Cells were seeded into 96-well cell culture plates (Beaver Biomedical Engineering Co., Ltd, 40196, Suzhou, Jiangsu province, China) at 3 × 10^3^ cells/well and allowed to adhere for 24 h. Then, cells were treated with different concentrations of AKBA (0~100 μm) for 24, 48, and 72 h. 10 μL CCK-8 (Beyotime Biotechnology, C0037) was added to the wells after treatment and incubated at 37°C for 1 h. The absorbance of the measure was measured at 450 nm by SpectraMax M5 spectrophotometer (Molecular Devices, Sunnyvale, CA). The viability of human colon cancer cells after treatment of AKBA was expressed as the proportion of optical density (OD) compared with that of the control (untreated cells) and IC_50_ values were calculated using GraphPad Prism 8.0 software (GraphPad Software Inc., USA).

- 1. **EdU-DNA synthesis assay**

DNA synthesis activity in cells treated by AKBA was checked using the EdU (5-ethynyl-2′-deoxyuridine, EdU)-DNA synthesis assay. HCT116 and SW620 Cells were seeded in 96-well plates at 1 × 10^4^ cells/well. After incubation of 24 h, 0, 20, 30, and 40 μm AKBA were added to the cells for 24 h, respectively. Cell-Light EdU Apollo 567 *In Vitro* kit (RIBOBIO Co. Ltd, C10310-1, Guangzhou, Guangdong province, China) was used according to the supplier’s protocol. 50 μM of EdU solution was then added to the cells and incubated for 2 h. After washed with PBS, the cells were fixed in 4 % paraformaldehyde for 20 min, and permeated with 0.5 % Triton X-100 (Solarbio Life Science, T8200) for 10 min. Next, an Apollo stain reaction solution was added for 30 min. Finally, cells were counter-stained with Hoechst 33342 (Solarbio Life Science, C0030) for 30 min and imaged by high content imaging system (Cellomics ArrayScan VTI, Thermo Fisher Scientific, Carlsbad, CA). The percentage of EdU positive cells in each field was using the following formula: Number of EdU positive cells/Total number of DAP stained cells. The data were performed in triplicate.

- 1. **Plate colony formation assay**

Plate cloning assay was used to evaluate the clone formation ability of individual cells. HCT 116 and SW620 cells were seeded into 6-well plates (Beaver Biomedical Engineering Co., Ltd, 40106) at 500 per well. After incubation of 24 h, 0, 20, 30, and 40 μM AKBA was added to the cells and incubated for 14 d, then the cells were fixed in 4 % paraformaldehyde (Solarbio Life Science, P1110) for 20 min, and stained with 0.1 % crystal violet solution (Solarbio Life Science, G1063) before being captured. The experiment was performed in triplicate.

- 1. **Cell cycle assay**

Propidium iodide (PI) is a fluorescent dye for double-stranded DNA and the fluorescence intensity is directly proportional to the content of the double-stranded DNA. The cells (2 × 10^6^) were seeded into the 6 cm culture dishes. After incubation for 12 h, the cells were treated with AKBA (20, 30, and 40 μM) for 24 h. The cells were washed with PBS (Solarbio Life Science, P1010) and harvested by using 0.25% trypsin-EDTA (Solarbio Life Science, T1300). Then, the cells were fixed in ice-cold 70% ethanol and stored at −4°C for 12 h. Fixed cells were centrifuged at 1000 rpm (Allegra X-22R, Beckman coulter, USA) for 5 min at room temperature and washed with PBS. Cells were stained with PI staining solution (50 μg/mL, Beyotime Biotechnology, ST511)for 30 min, and RNase A (20 μg/mL, Beyotime Biotechnology, ST578). A C6 flow cytometer (BD Accuri^TM^, Maryland, Baltimore, USA) was used to analyze the fluorescence output at 488 nm. FlowJo 10.8.1 software (BD Bioscience, Franklin Lake, New Jersey, USA) was used for data analysis.

### Table S1. Primer sequences.

| NO. | Primer | Species | Sequences 5' to 3' |
| --- | --- | --- | --- |
| 1 | IFN-γ | Mouse | F:TTGCCAAGTTTGAGGTCAACAA  R:CGCTTCCTGAGGCTGGATTC |
| 2 | IL-1β | Mouse | F:TGCCACCTTTTGACAGTGATG  R:TGATGTGCTGCTGCGAGATT |
| 3 | IL-6 | Mouse | F: TCCGGAGAGGAGACTTCACA  R: TTGCCATTGCACAACTCTTTTC |
| 4 | TNF-α | Mouse | F:GCAGCCTTGTCCCTTGAAGA  R:TTCTCAAAAGGACAGCCTCG |
| 5 | IL-10 | Mouse | F:GCATGGCCCAGAAATCAAGG  R:ACACCTTGGTCTTGGAGCTTATTA |
| 6 | β-actin | Mouse | F: GTCGT ACCAC AGGCA TTGTG ATGG  R:GCAAT GCCTG GGTAC ATGGT GG |

**
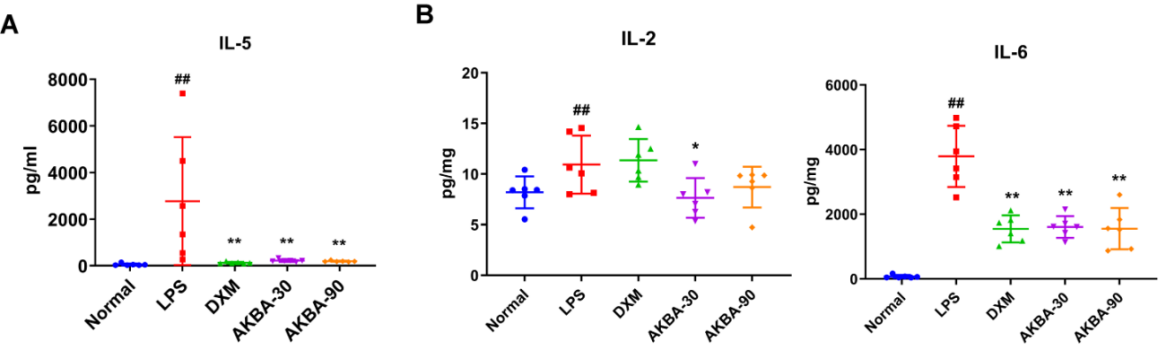
**

**Figure S4 Anti-inflammatory effect of AKBA on LPS-induced inflammation mice. A.** The serum level of IL-5 in serum for each group (n = 6). **B.** The serum levels of IL-2 and IL-6 for each group (n = 6). Note: Normal, normal control group; LPS, model group; DXM, (LPS + dexamethasone 5 mg/kg); AKBA-30, (LPS + AKBA 30 mg/kg); AKBA-90, (LPS + AKBA 90 mg/kg); and 5-Fu, (AOM/DSS+5-Fu, 20 mg/kg). Note: ##*p*<0.01, compared with normal control group; **p*<0.05, ***p*<0.01, compared with LPS group.


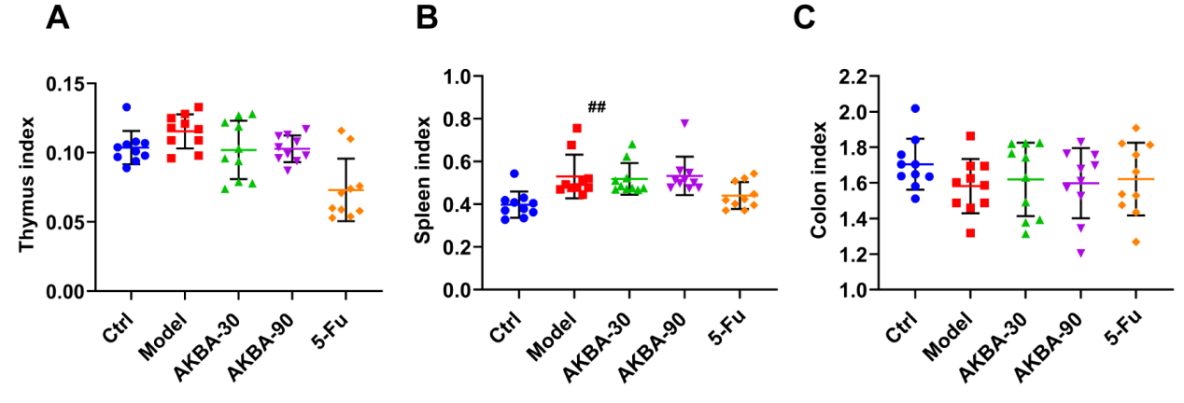


**Figure S5. The effect of AKBA on CRC mice induced by AOM/DSS**. **A.** Thymus index for each group (n = 10); **B.** Spleen index for each group (n = 10); **C.** Colon index for each group (n = 10). Note: Ctrl., Normal control group; Model, AOM/DSS induced group; AKBA-30, (AOM/DSS+AKBA 30 mg/kg); AKBA-90, (AOM/DSS+AKBA 90 mg/kg) and 5-Fu, (AOM/DSS+5-Fu, 20 mg/kg). Note: ##*p*<0.01, compared with Ctrl. group.

**Table S2. Primary antibody information and the dilution ratio in western blot.**

| NO. | Name | Manufacture | Cat | City | MW  (kDa) | Dilution |
| --- | --- | --- | --- | --- | --- | --- |
| 1 | Anti-IKK alpha + IKK beta (phospho S176 + S177) antibody | Abcam | ab194528 | Boston, Massachusetts, USA | 80/85 | 1：1000 |
| 2 | anti-IKKα antibody | Abcam | ab32041 | Boston, Massachusetts, USA | 85 | 1:1000 |
| 3 | anti-IκBα (L35A5) Mouse mAb (Amino-terminal Antigen) | Cell signaling technology, | 4814S | Danvers, Massachusetts, USA | 39 | 1:1000 |
| 4 | anti-Phospho-IκBα (Ser32/36) (5A5) Mouse mAb | Cell signaling technology | 9246S | Danvers, Massachusetts, USA | 40 | 1:1000 |
| 5 | anti-NF-κB p65 (D14E12) XP® Rabbit mAb | Cell signaling technology | 8242T | Danvers, Massachusetts, USA | 65 | 1:1000 |
| 6 | anti-Phospho-NF-κB p65 (Ser536) (93H1) Rabbit mAb | Cell signaling technology | 3033T | Danvers, Massachusetts, USA | 65 | 1:1000 |
| 7 | anti-GAPDH antibody | Proteintech group Inc. | 60004-1-1g | Chicago, Illinois, USA | 36 | 1:2000 |

### Table S3. The Enrich KEGG pathways (top20).

| Term | Gene name |
| --- | --- |
| Ribosome | RPL17/RPL28/RPS14/RPS10/RPL18/RPL15/MRPL1/RPL13/RPL36/RPS6/RPL4/RPS5/UBA52/RPS28/RPL14/RPL29/RPS13/RPS25/FAU/RPL24/RPL7A/MRPS18C/RPS15/RPL19/RPL3/RPL18A/RPS7/RPL21/RPL10/RPL37/RPL23A/MRPS15/RPS27/RPS19/RPS16/RPL38/RPS3/RPL11/RPL27A/RPS12/RPL35A/RPL32/RPL7/RPL6/RPL12/MRPL34/RPLP0/RPL35/RPS20/RPL13A/RPS15A/RPL39/RPL10A/RPL37A/RPL30/RPL31/RPL8/RPL34/RPS8/RPLP2/MRPL23/RPS11 |
| Cell cycle | BUB1/CDK1/E2F2/ORC6/CCNB1/BUB1B/CCNA2/MAD2L1/GADD45B/TTK/CDC25C/PLK1/PKMYT1/CCNB2/RBL1/SFN/MCM4/ESPL1/CDC45/CDC7/MYC/E2F1/CCNE2/GADD45A/ORC1/MCM2/CDKN2C/PTTG1/CDC25A/MCM3/PCNA/CCNB3/YWHAB/TFDP1/SMC1A/GADD45G/CDKN2D/WEE1/RB1/CDC20/SKP2/CDC6/CUL1/CDKN1A/CCNE1/CHEK2/CHEK1/PRKDC/YWHAH/CDC27/CDC14B/HDAC1/ATR |
| NF-kappa B signaling pathway | CXCL8/TNFAIP3/BIRC3/GADD45B/NFKBIA/RELB/CXCL2/TIRAP/RIPK1/BCL2/NFKB2/SYK/TRAF1/TRIM25/MYD88/DDX58/CSNK2A1/CSNK2A3/ICAM1/LYN/TICAM1/PRKCQ/PARP1 |
| IL-17 signaling pathway | CXCL8/TNFAIP3/MMP1/CSF2/CXCL3/NFKBIA/CXCL1/FOS/FOSL1/JUN/CXCL2/FOSB/TRAF3IP2/FADD/IL17RB/IL17RE/CEBPB/CASP8/JUND/S100A7/HSP90AB1 |
| DNA replication | FEN1/MCM4/POLE2/MCM2/PRIM1/POLD3/POLE/MCM3/PCNA/POLA2/POLA1/RFC5/RNASEH2B/LIG1/PRIM2/DNA2/RFC3/RPA3/RFC4/POLD4/RFC2 |
| Steroid biosynthesis | MSMO1/CYP24A1/SQLE/DHCR7/FDFT1/CYP51A1/EBP/HSD17B7/NSDHL/DHCR24/CYP2R1/SC5D |
| Cellular senescence | CXCL8/CDK1/E2F2/CCNB1/CCNA2/MYBL2/GADD45B/FOXM1/CCNB2/RBL1/MYC/E2F1/CCNE2/GADD45A/RASSF5/ETS1/CDC25A/MAP2K3/LIN54/CCNB3/RRAS/GADD45G/PPP3CA/CAPN2/SERPINE1/FOXO3/RB1/PIK3CD/CDKN1A/TRAF3IP2/NBN/IGFBP3/BTRC/CCNE1/IL1A/CHEK2/CHEK1/VDAC2/PPP1CC/MAP2K2/SQSTM1/SLC25A4/RAD9A/SLC25A6/NFATC2/HUS1/ATR |
| p53 signaling pathway | CDK1/RRM2/CCNB1/GADD45B/GTSE1/CCNB2/SFN/BBC3/TP73/CCNE2/GADD45A/ADGRB1/BCL2/SESN2/GADD45G/SERPINE1/CDKN1A/SESN3/IGFBP3/CCNE1/THBS1/TNFRSF10B/CHEK2/CHEK1/CASP8/APAF1/ATR |
| Nucleotide excision repair | POLE2/POLD3/POLE/PCNA/RFC5/GTF2H3/LIG1/ERCC4/RFC3/RPA3/GTF2H2C/RFC4/GTF2H5/POLD4/XPA/RFC2 |
| Bladder cancer | CXCL8/MMP1/E2F2/HBEGF/MYC/E2F1/DAPK3/RB1/CDKN1A/THBS1/MAP2K2/MMP2/TYMP/RASSF1/EGF |
| Transcriptional misregulation in cancer | CXCL8/BIRC3/GADD45B/CSF2/MYC/TFE3/GADD45A/CDKN2C/ETV1/PBX1/NFKBIZ/DDIT3/HIST1H3H/NTRK1/NGFR/ITGAM/ETV6/GADD45G/ZEB1/PPARG/ARNT2/HMGA2/CDKN1A/TRAF1/IGFBP3/FUS/MEF2C/SIX1/SMAD1/DOT1L/CEBPB/PDGFA/HIST1H3E/RARA/NR4A3/BAK1/MET/REL/NUPR1/HDAC1/PML |
| Small cell lung cancer | E2F2/BIRC3/GADD45B/NFKBIA/MYC/LAMB3/E2F1/CCNE2/GADD45A/ITGA2/FN1/BCL2/GADD45G/RB1/PIK3CD/SKP2/CDKN1A/TRAF1/CCNE1/COL4A6/LAMA5/LAMA3/LAMA4/ITGAV/LAMC3/APAF1/BAK1 |
| Apoptosis | BIRC5/BIRC3/GADD45B/ACTG1/ACTB/NFKBIA/FOS/BBC3/LMNB1/GADD45A/PRF1/CTSO/JUN/DDIT3/RIPK1/BCL2/NTRK1/DIABLO/GADD45G/CTSV/CAPN2/PIK3CD/TRAF1/FADD/ATF4/TNFRSF10B/MCL1/PARP4/AIFM1/CTSK/MAP2K2/CASP8/APAF1/BAK1/PARP1/PTPN13 |
| Hepatitis B | CXCL8/BIRC5/E2F2/CCNA2/NFKBIA/FOS/CREB5/MYC/E2F1/CCNE2/JUN/TIRAP/PCNA/BCL2/YWHAB/STAT1/TLR3/RB1/PIK3CD/CDKN1A/FADD/CCNE1/ATF4/MYD88/DDX58/EGR2/IRF7/PTK2B/MAP2K2/TICAM1/IFIH1/CASP8/APAF1/NFATC2/ATP6AP1 |
| NOD-like receptor signaling pathway | CXCL8/TNFAIP3/BIRC3/CXCL3/NFKBIA/CXCL1/GBP2/JUN/CXCL2/NLRP1/OAS3/RHOA/RIPK1/BCL2/RIPK2/ANTXR1/TMEM173/STAT1/MFN1/BRCC3/FADD/GABARAPL1/PLCB4/MYD88/IRF7/VDAC2/NLRP12/CARD9/PLCB1/ERBIN/RNF31/TICAM1/CASP8/NLRC4/PANX1/NFKBIB/NLRP6/HSP90AB1 |
| MicroRNAs in cancer | E2F2/CDCA5/BRCA1/CDC25C/SOCS1/CYP24A1/PIM1/MYC/E2F1/CCNE2/KIF23/IRS2/HMOX1/HDAC4/IRS1/CDC25A/RHOA/SOS1/BCL2/SOX4/ZEB1/DDIT4/DNMT1/SHC4/HMGA2/FZD3/CYP1B1/MARCKS/CDKN1A/ZFPM2/WNT3A/FSCN1/CCNE1/THBS1/MCL1/DNMT3B/RPTOR/MAP2K2/PDGFA/TNC/BAK1/STMN1/MET/HDAC1/RASSF1 |
| Rheumatoid arthritis | CXCL8/MMP1/CSF2/CXCL1/FOS/ATP6V0D2/IL23A/JUN/IL11/ATP6V1E2/IL1A/CTSK/ICAM1/IL15/ACP5/ATP6AP1 |
| Toll-like receptor signaling pathway | CXCL8/IL12A/NFKBIA/FOS/JUN/TIRAP/MAP2K3/RIPK1/STAT1/TLR3/PIK3CD/TLR1/FADD/MYD88/IRF7/CTSK/MAP2K2/TLR6/TLR5/TICAM1/CASP8 |
| Breast cancer | E2F2/HES1/BRCA2/BRCA1/GADD45B/FOS/MYC/E2F1/GADD45A/JUN/FZD4/SOS1/WNT7A/FGF8/GADD45G/NFKB2/FGFR1/SHC4/FZD3/RB1/PIK3CD/DVL1/CDKN1A/WNT3A/DLL3/FGF9/HEY2/MAP2K2/FRAT2/BAK1/SHC2/HEY1/EGF |
| Regulation of actin cytoskeleton | ACTG1/ACTB/DIAPH3/IQGAP3/RAC2/ITGA2/FN1/LPAR5/RHOA/SOS1/ITGB8/RRAS/ARHGEF6/ITGAM/FGF8/SSH1/ROCK2/ITGAX/WASF2/FGFR1/MYL12A/PFN1/MYH9/PIK3CD/BAIAP2/ITGB5/MYH14/FGF9/MYL9/CDC42/TIAM1/MYLK2/LPAR1/PPP1CC/LPAR2/ACTN1/MAP2K2/GNA13/MYH10/PDGFA/ARHGEF4/ITGAV/ITGAD/IQGAP2/SLC9A1/EGF/MYLK |

### Table S4. Intestinal flora diversity index of three groups.

| Index | Con. | Mod. | AKBA |
| --- | --- | --- | --- |
| shannon | 4.07±0.18 | 3.49±0.35 | 4.04±0.18 |
| chao | 3.92±0.28 | 3.81±0.57 | 4.04±0.18 |
| coverage | 0.99±0.00 | 0.99±0.00 | 0.99±0.00 |
